# Dehydrogenation of formic acid by Ir–bisMETAMORPhos complexes: experimental and computational insight into the role of a cooperative ligand†
†Electronic supplementary information (ESI) available. CCDC 1020151. For ESI and crystallographic data in CIF or other electronic format see DOI: 10.1039/c4sc02555e



**DOI:** 10.1039/c4sc02555e

**Published:** 2014-10-22

**Authors:** Sander Oldenhof, Martin Lutz, Bas de Bruin, Jarl Ivar van der Vlugt, Joost N. H. Reek

**Affiliations:** a van ’t Hoff Institute for Molecular Sciences , University of Amsterdam , Science Park 904 , 1098 XH , Amsterdam , The Netherlands . Email: j.n.h.reek@uva.nl; b Bijvoet Center for Biomolecular Research , Utrecht University , Padualaan 8 , 3584 CH , Utrecht , The Netherlands

## Abstract

The synthesis and tautomeric nature of three xanthene-based bisMETAMORPhos ligands (**La–Lc**) is reported. Coordination of these bis(sulfonamidophosphines) to Ir(acac)(cod) initially leads to the formation of Ir^I^(**L** ^H^) species (**1a**), which convert *via* formal oxidative addition of the ligand to Ir^III^(**L**) monohydride complexes **2a–c**. The rate for this step strongly depends on the ligand employed. Ir^III^ complexes **2a–c** were applied in the base-free dehydrogenation of formic acid, reaching turnover frequencies of 3090, 877 and 1791 h^–1^, respectively. The dual role of the ligand in the mechanism of the dehydrogenation reaction was studied by ^1^H and ^31^P NMR spectroscopy and DFT calculations. Besides functioning as an internal base, bisMETAMORPhos also assists in the pre-assembly of formic acid within the Ir-coordination sphere and aids in stabilizing the rate-determining transition state through hydrogen-bonding.

## Abstract 2

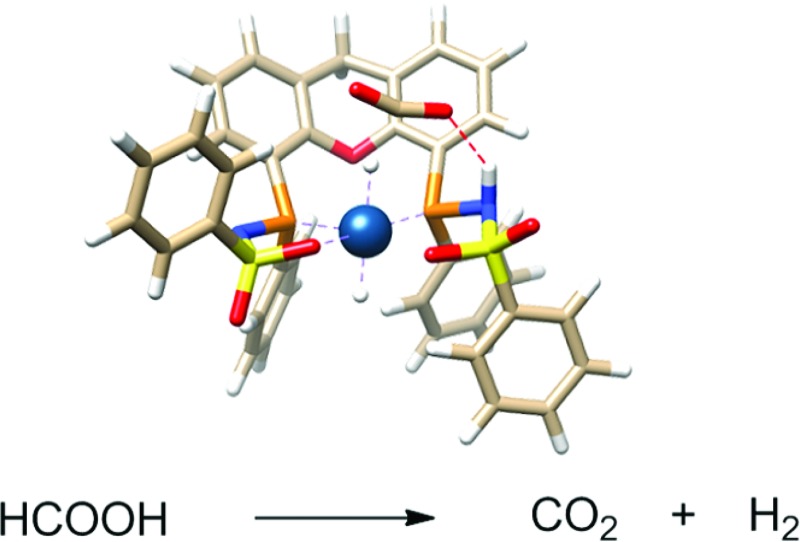
The synthesis of Ir-complexes with three bisMETAMORPhos ligands is reported. The activity of these systems towards HCOOH dehydrogenation and the dual role of the ligand during catalysis is discussed, using spectroscopic and computational methods.

## Introduction

Enzyme active sites are a major source of inspiration for scientists in the field of synthetic chemistry and homogeneous catalysis, because of the high activities and selectivities achieved in chemical transformations and the conceptual strategies employed by these systems.^1^ For instance, weak but highly directional hydrogen bonding stands out as a key element used by enzymes to selectively pre-assemble and pre-activate substrates and stabilize transition states. Inspired by this, functional ligands decorated with H-bond donor and/or acceptor groups have been used to construct supramolecular ligand structures, enabling modular ligand families^2^ to be utilized in transition metal catalysis and also for substrate pre-organization and pre-activation *via* specific ligand–substrate interactions.^3^

**Fig. 1:**
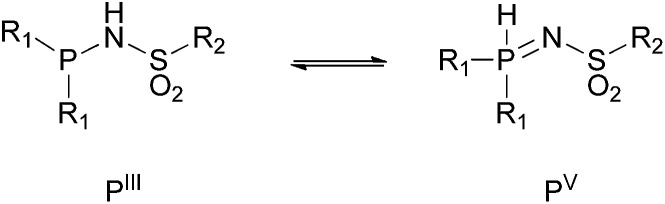
General composition of METAMORPhos ligands with their two tautomeric forms [N*H*–P (P^III^) and N=P*H* (P^V^)] in equilibrium.

In our quest for novel systems able to undergo hydrogen-bonding interactions to steer reactivity we have developed sulfonamidophosphine (METAMORPhos) ligands. These ligands are based on a PNSO_2_ scaffold that displays N*H*–P/N=P*H* tautomerism (see Fig. 1). They have been employed for mono- and bimetallic Rh-catalyzed hydrogenation, Ru-based heterolytic H_2_ cleavage and [2 + 2 + 2] cycloaddition reactions.^4^

**Fig. 2:**
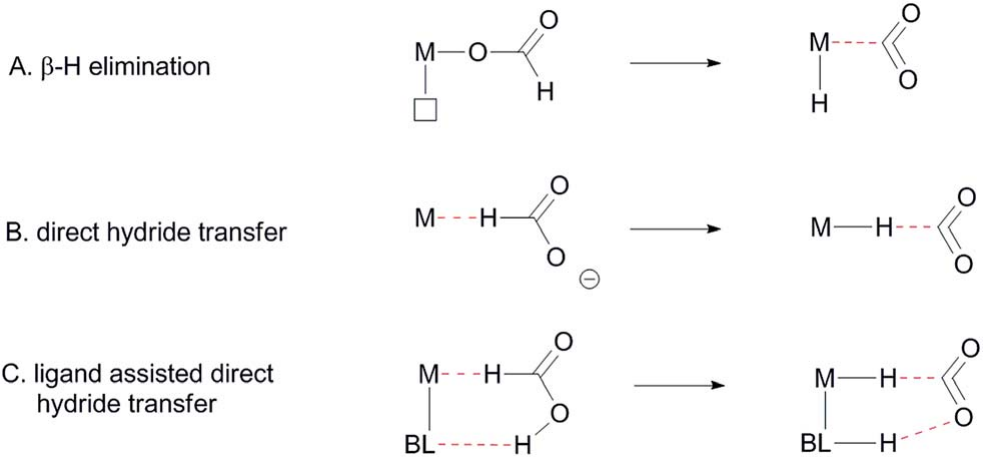
Different proposed mechanisms for the dehydrogenation of formic acid, BL: bifunctional ligand.

Formate dehydrogenase metalloenzymes have successfully been employed for the reduction of CO_2_ and oxidation of formate. Although there is no complete consensus concerning the mechanism of either the reduction or the oxidation, hydrogen-bonding and proton-shuttling are suggested to play a crucial role in the way in which these enzymes operate.^5^ We recently described our initial results with an Ir-catalyst bearing a bisMETAMORPhos ligand in the base-free catalytic dehydrogenation of formic acid, a reaction that has attracted much recent interest in the context of hydrogen storage/release systems.^4*f*^ Typically, formic acid (HCOOH) dehydrogenation catalysts require the addition of sub-stoichiometric amounts of base (*e.g.* 5 : 2 ratio of HCOOH : NEt_3_). However, this significantly reduces the overall hydrogen weight percentage of the reaction mixture.^6^ One promising strategy to circumvent the use of exogenous base is to employ catalyst systems bearing cooperative ligands to access metal–ligand bifunctional pathways for substrate activation.^7^ Hydrogen-bonding interactions between ligand and substrate as (additional) tools to enhance reactivity have previously been proposed both in the hydrogenation of CO_2_ and in the aluminium catalysed dehydrogenation of HCOOH.^8^ These interactions were also suggested to play a role in the formation of half-sandwich ruthenium and rhodium hydrides from formic acid.^9^ Several studies on the mechanism of HCOOH dehydrogenation have compared conventional β-hydride elimination with direct hydride-transfer (Fig. 2A and B).^10^ Given the potential H-bonding abilities of METAMORPhos ligands and the protic nature of formic acid, we wondered if biomimetic non-covalent interactions between the ligand backbone and formic acid could play a role in this catalytic system. Herein we show that the proton-responsive ligand not only acts as an internal base (Fig. 2C), but that its hydrogen-bonding abilities steer substrate pre-assembly and stabilize catalytic transition states.

We will present the synthesis of three bisMETAMORPhos ligands as well as their coordination to iridium, compare the activity of these systems in HCOOH dehydrogenation and explain the dual role of the ligand framework during catalysis by means of both experimental and computational results.

## Results and discussion

### Synthesis of ligands and complexes

**Scheme 1:**
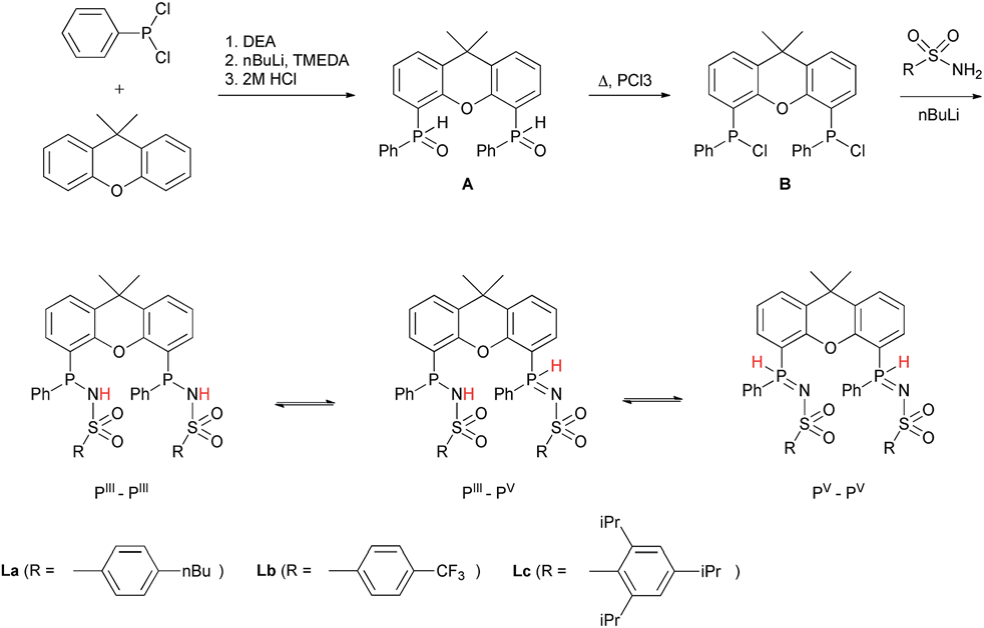
The synthesis of bisMETAMORPhos ligands **La–Lc** and their three tautomeric forms (P^III^–P^III^, P^III^–P^V^ and P^V^–P^V^).

METAMORPhos ligands are prepared by a simple condensation reaction between a sulfonamide of choice and a chlorophosphine. They show high stability to both oxidation (at phosphorus) and hydrolysis (of the P–N bond). We attribute this stability to their prototropic character, resulting in an equilibrium between the P^III^ and P^V^ oxidation states that is influenced by R_1_ and R_2_.^4*e*^ In order to gain insight into the effect of ligand modification on the coordination and degree of tuning in HCOOH dehydrogenation catalysis, we prepared bisMETAMORPhos ligands **La–Lc** *via* a three-step synthetic protocol (Scheme 1) that results in selective formation of only the respective *meso*-isomers, see ESI.†.

All three ligands **La–Lc** display P^III^/P^V^ tautomerism, but in different ratios for the three possible forms. For ligand **La** only the P^III^–P^III^ and P^III^–P^V^ tautomers were observed in CD_2_Cl_2_ in a ratio of 1 : 0.4, as determined by ^31^P NMR spectroscopy. For both ligands **Lb** and **Lc** all three possible tautomers were observed, with ratios (P^III^–P^III^ : P^III^–P^V^ : P^V^–P^V^) of 1 : 1.8 : 0.2 and 1 : 1.9 : 0.1 for **Lb** and **Lc**, respectively. These data show that the overall P^III^/P^V^ ratio is not only determined by the acidity of the N–H bond and basicity of the phosphorus atom but is also greatly influenced by the steric bulk of the substituent on the sulfon group. The P^V^ tautomer provides stability towards oxidation and hydrolysis, even to the extent that **La–Lc** can be conveniently purified by column chromatography. Upon coordination to a metal center, the tautomeric behaviour of these ligands is lost.

**Scheme 2:**
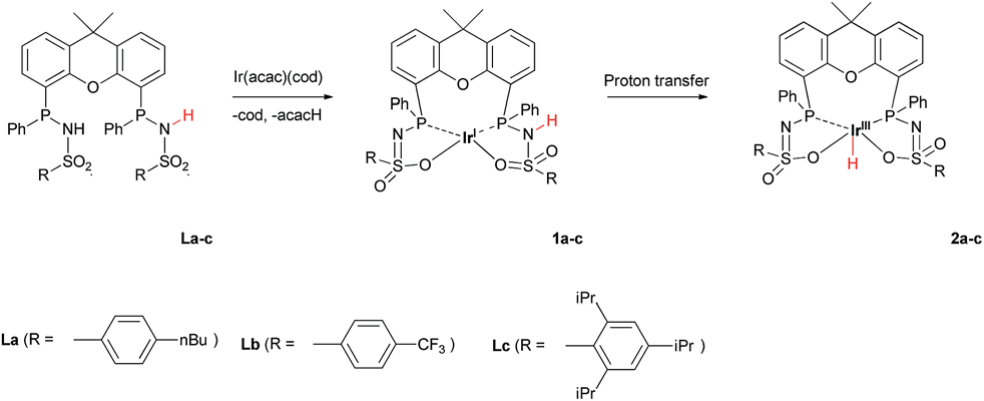
Synthesis of complexes **1a–c** and **2a–c** from Ir^I^(acac)(cod) and **La–c**.

The addition of **La–Lc** to Ir^I^(acac)(cod) generated complexes [Ir^I^(**L** ^H^)] **1a–c** *via* a single proton-transfer from the ligand to acetylacetonate and displacement of cyclooctadiene. These species show symmetric ^1^H and ^31^P NMR spectra, irrespective of the specific ligand substitution pattern, which likely originates from highly fluxional behaviour between the protonated and deprotonated ligand arms. Formal oxidative addition of the remaining ligand –NH group in **1a–1c** generates the corresponding Ir^III^(H)(**L**) complexes **2a–c** (Scheme 2), see also ESI.†

**Fig. 3:**
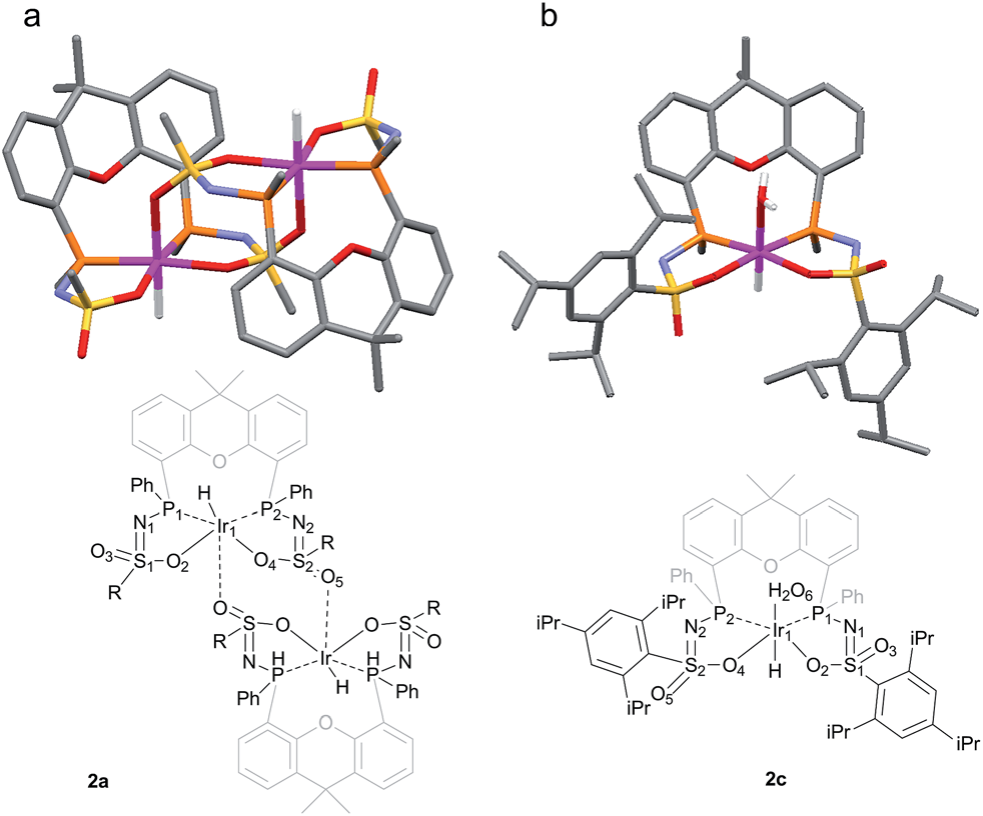
(a) Schematic representation and molecular structure of **2a**.^4*f*^ (b) Schematic representation and molecular structure of **2c**.^14^ Solvent molecules and H-atoms omitted for clarity, except for hydride and hydrogens of the aqua ligand. Color labels: purple: iridium, orange: phosphorus, blue: nitrogen, yellow: sulfur, red: oxygen. Selected bond lengths (Å) and angles (°) for **2c**: N_1_–S_1_ 1.554(2), N_2_–S_2_ 1.554(2), S_1_–O_2_ 1.4974(18), S_2_–O_4_ 1.5093(18), S_1_–O_3_ 1.4541(18), S_2_–O_5_ 1.439(2), Ir_1_–P_1_ 2.2311(6), Ir_1_–P_2_ 2.2315(6), Ir_1_–O_2_ 2.1639(18), Ir_1_–O_4_ 2.1752(17), Ir_1_–O_6_ (H_2_O) 2.2427(18), P_1_–Ir_1_–P_2_ 104.54(2).

The rate for this overall proton-transfer step varies significantly for ligands **1a–c**. Ir^III^ complex **2a** (with **La**) was obtained quantitatively after 30 hours at room temperature, but no formation of complex **2b** was observed under the same conditions. This species could only be obtained after heating the mixture for 40 hours at 70 °C. In contrast, the conversion of **1c** into **2c** was significantly faster than the conversion of **1a** into **2a**, and full conversion was already observed after 16 hours at room temperature. We previously reported the molecular structure of **2a** to be dimeric in the solid state (**2a_2_**), with the ‘vacant’ axial site coordinated by an oxygen from the sulfonamide of a second equivalent of **2a** (Fig. 3a).^4*f*^ The molecular structure determination for the more bulky analogue **2c** revealed a slightly distorted octahedral mononuclear Ir^III^ hydride complex, with axial coordination of a water molecule *trans* to the hydride to complete the octahedral coordination environment of Ir^III^ (see Fig. 3b). The steric hindrance of the isopropyl groups in complex **2c** seems to effectively prevent the formation of dinuclear complexes, as was also found by modelling studies. This finding also supports the previously proposed mononuclear configuration in solution, based on diffusion NMR data.^4*f*^ The N–S bond lengths of 1.554(2) Å are in agreement with deprotonated sulfonamide fragments.^4*f*^ The Ir–O_water_ bond length was found to be 2.2427(18) Å, which is in accordance with *trans*-Ir^III^(H)(OH_2_) complexes described in literature.^11^

### Dehydrogenation studies with complexes **2a–c**

**Fig. 4:**
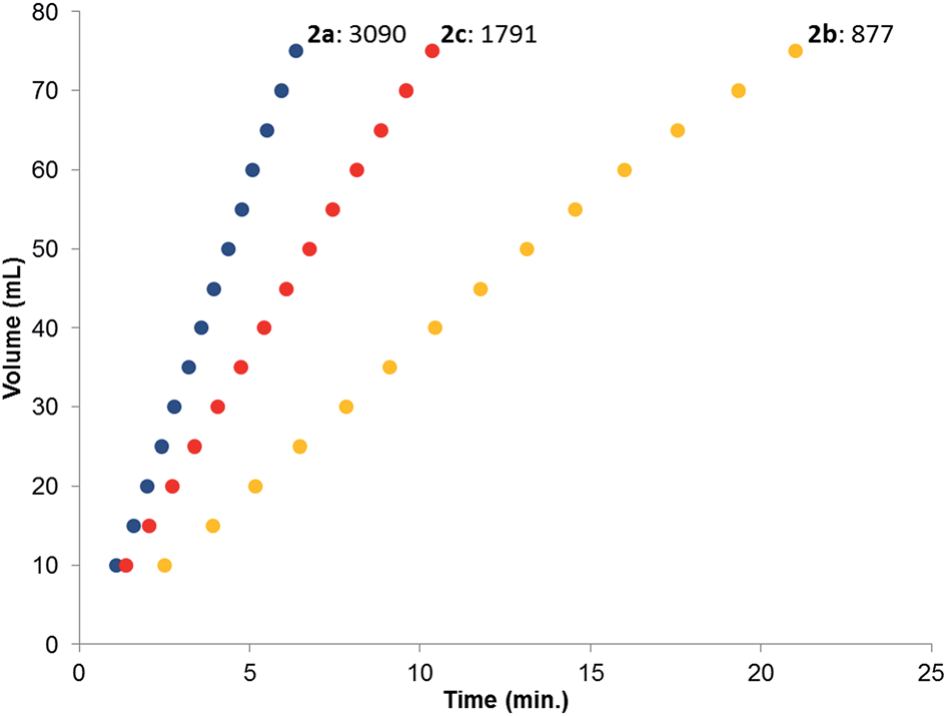
Dehydrogenation curves of **2a–c** (TOFs are determined between 4–30% conversion); total measured volume (mL) consists of both H_2_ and CO_2_.

**Fig. 5:**
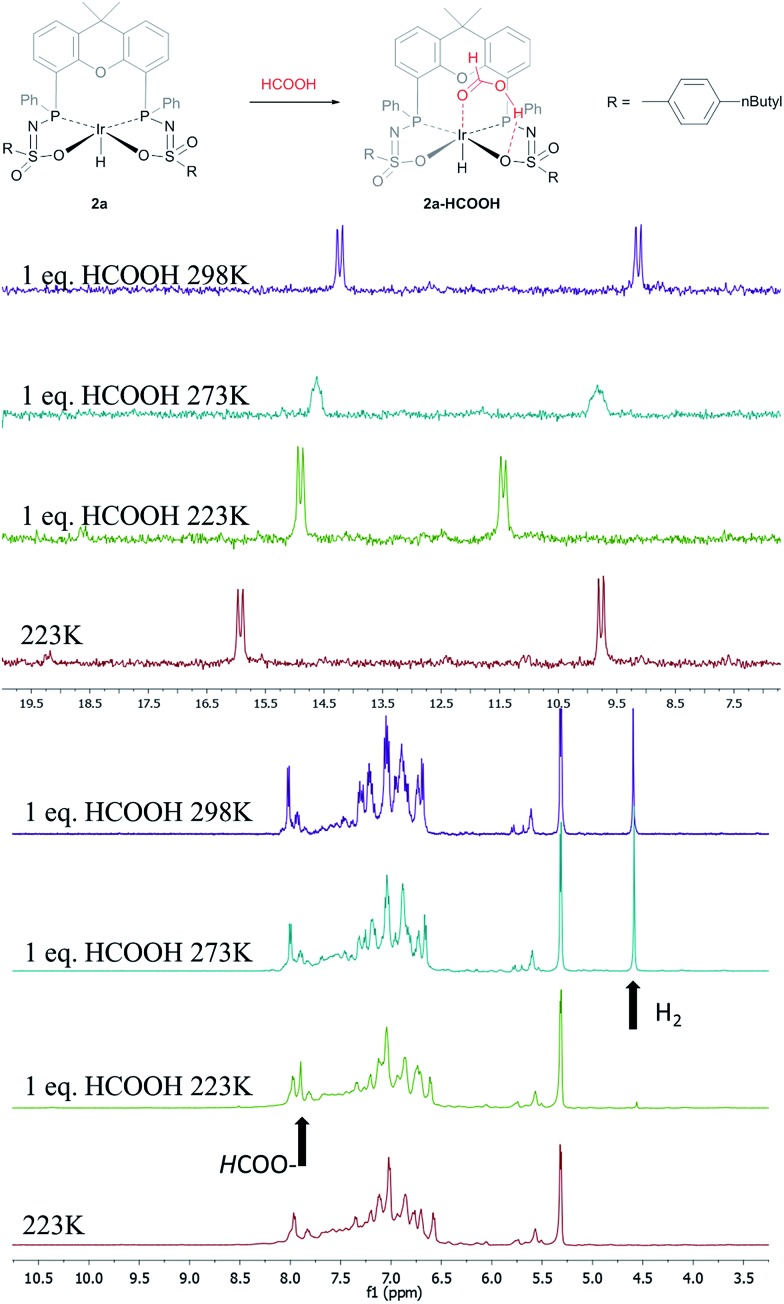
Proposed coordination of HCOOH to complexes **2** to form a **2a**–HCOOH adduct (top). VT ^31^P NMR spectra of **2a** with one equivalent of HCOOH (left). VT ^1^H NMR spectra of **2a** with one equivalent of HCOOH; arrows indicate HCOO–proton and H_2_ formed (right).

The catalytic dehydrogenation of HCOOH was investigated with complexes **2a–c**.‡
‡Dehydrogenation studies were performed with diastereomeric mixtures of complexes **2a–c** (upon anionic coordination of the sulfon group these become chiral and give rise to diastereomeric mixtures of the complexes **2**), see ESI† and ref. 4f for more information. Catalysis was performed in toluene at 85 °C in the absence of base to generate dehydrogenation curves that are shown in Fig. 4, see also ESI.† The turnover frequencies (TOFs) of 3090 (**2a**), 877 (**2b**) and 1791 h^–1^ (**2c**) reveal a correlation with the electronic nature of the sulfonamide organic side-group, *i.e.* high activity is obtained with an electron-donating group in the *para*-position (**2a**, *n*-butyl), whereas an electron-withdrawing group in the *para*-position (**2b**, CF_3_) results in much lower activity (see Computational section for explanation). Also with sterically encumbered complex **2c** a lower activity was obtained than with **2a**. Variable temperature (VT) NMR spectroscopy was performed to obtain mechanistic insight and detect relevant intermediates.§
§VT-NMR studies were performed with a diastereomerically pure form of **2a**; see ref. 4f for additional information. The addition of an equimolar amount of HCOOH to complex **2a** at 223 K led to a significant downfield shift (1.7 ppm) of one of the phosphorus signals (see Fig. 5, top). This might indicate (partial) protonation of one of the ligand arms. Upon increasing the temperature to 298 K the original ^31^P NMR spectrum for species **2a** is instantaneously restored with no observation of any intermediates.¶
¶The ^31^P NMR chemical shifts of **2a** are significantly affected by the temperature, but these changes are completely reversible, see ESI.†
 Addition of one equivalent of HCOOH to **2a** results in an upfield shift in the ^1^H NMR spectrum for the formate proton of 0.13 ppm at 223 K (Fig. 5, right) compared to free HCOOH, which is an indication of HCOOH coordination to the axial vacant site (Fig. 5, bottom). Increasing the temperature leads to decreasing HCOOH signals together with the formation of H_2_.

### Computational investigation into β-hydride elimination

**Fig. 6:**
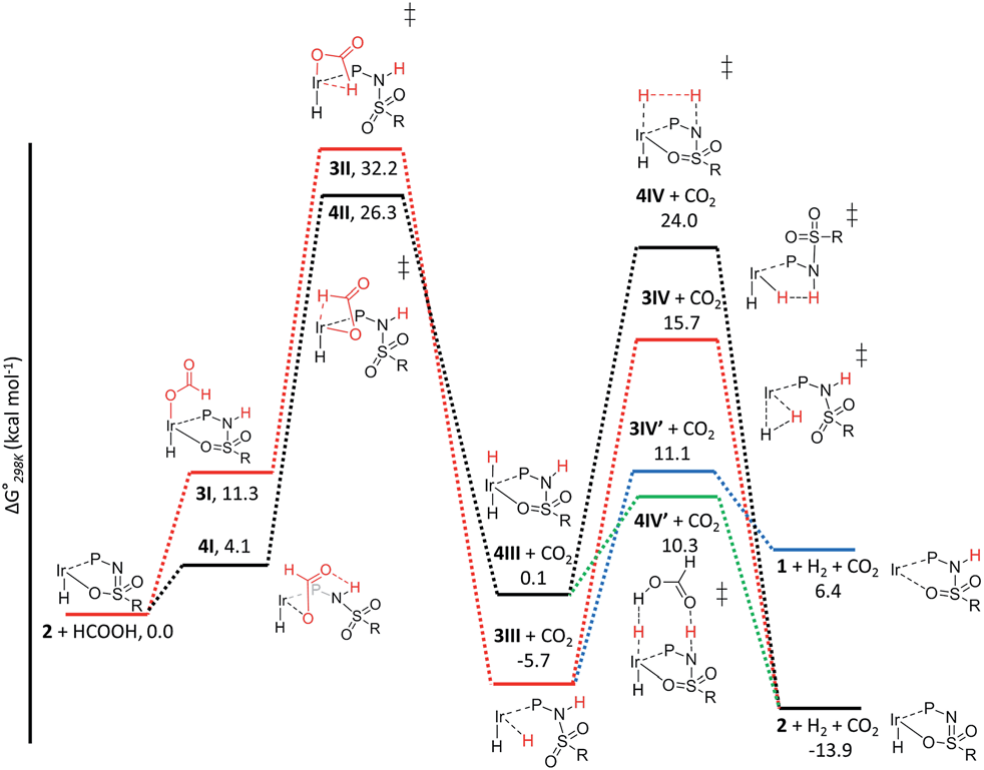
Potential energy diagram (DFT, BP86, def2-TZVP) for the dehydrogenation of formic acid *via* β-hydride elimination at the equatorial (red) and axial (black) positions; Δ*G*0298K is in kcal mol^–1^.

The C–H bond cleavage in the dehydrogenation of HCOOH, typically the rate determining step in the catalytic cycle, can either occur *via* β-hydride elimination or *via* (ligand-assisted) direct hydride-transfer of the formate hydrogen (*H*COOH) atom (see Fig. 2A–C). Both possible mechanisms were computationally investigated using DFT in order to shed light on the potential role of the proton-responsive bisMETAMORPhos ligand. The obtained energy profiles for β-hydride elimination towards an equatorial and an axial coordination site of the catalyst are shown in Fig. 6 (only the relevant part of the computed structures is shown).‖
‖All calculations were performed with R = phenyl on the sulfon group for computational simplicity using the diastereomeric form that was utilized in VT NMR studies and obtained as a crystal structure.


The first step in the energy profile towards equatorial β-hydride elimination (red profile) is complete proton-transfer of HCOOH to the cooperative ligand, resulting in the formation of κ^1^-formate complex **3I**, which is endergonic by 11.3 kcal mol^–1^. In this structure the formate C–H bond is pre-organized for β-hydride elimination *via* high barrier transition state **3II** (Δ*G* ^‡^ = 32.2 kcal mol^–1^).**
**Note that these are gas-phase calculations for which the translational entropy contributions are much larger than in solution. By calculation of the partition function of the molecules in the gas phase, the entropy of dissociation or coordination for reactions in solution is overestimated. Therefore, for reactions in solution, the Gibbs free energies for all steps involving a change in the number of species should be corrected. Several methods have been proposed for the correction of gas phase to solution phase data. The minimal correction term is a correction for the condensed phase (CP) reference volume (1 L mol^–1^) compared to the gas phase (GP) reference volume (24.5 L mol^–1^). This leads to an entropy correction term (SCP = SGP + R ln{1/24.5}) for all species, lowering the relative free energies (298 K) of all associative steps by 2.5 kcal mol^–1^.^3k,15^ According to some authors, this correction term is too small, and larger correction terms up to 6.0 kcal mol^–1^ have been suggested.^16^ Which correction term is best remains somewhat debatable. This produces *cis*-dihydride structure **3III**, which is an overall exergonic process (–5.7 kcal mol^–1^). Structure **3III** can release H_2_ *via* protonation of an iridium hydride by the ligand (N–H or O–H) or reductive elimination of the two hydride units. Protonation of the hydride by the N–H moiety of the ligand in transition state **3IV** has a rather high barrier (red profile, 21.4 kcal mol^–1^). No transition state was found for protonation of the hydride *via* an O–H group. Transition state **3IV** is potentially stabilized by axial coordination of the ligand *via* the sulfon group, which is in proximity to the iridium (2.42 Å). This stabilization cannot occur upon protonation *via* the O–H moiety, which potentially explains why no transition state could be found. The reductive elimination of H_2_ forms Ir^I^ structure **1** (similar to the initially formed complexes **1a–c** that lead to the formation of **2a–c**) *via* transition state **3IV′**, which is 4.6 kcal mol^–1^ lower in energy (blue energy profile, 11.1 kcal mol^–1^) than **3IV**. After release of H_2_ the formed structure **1** is 6.4 kcal mol^–1^ higher in energy than the starting structure **2**. This is in agreement with experimental observations, as iridium(i) complexes **1a–c** eventually transform to the thermodynamically more stable iridium(iii)-hydride complexes **2a–c**. The above described pathway to *cis*-dihydride species **3III** *via* transition state **3II** (Fig. 6, red line) seems unlikely to occur, as the activation barrier obtained for C–H cleavage is rather high (32.2 kcal mol^–1^). The β-hydride elimination towards the axial position was found to be energetically more favorable (Fig. 6, black energy profile). Deprotonation of HCOOH by the ligand and formation of κ^1^-formate structure **4I** is endergonic by 4.1 kcal mol^–1^. The protonated ligand showed a N–H···O hydrogen bonding interaction between the ligand and the coordinated formate. β-Hydride elimination from **4I** *via* transition state **4II** to yield **4III** has an activation barrier of 26.3 kcal mol^–1^. The *trans*-dihydride structure **4III** is slightly endergonic by 0.1 kcal mol^–1^. Protonation of the Ir–H bond by a ligand N–H group to release H_2_ from **4III** *via* transition state **4IV** has a high barrier of 24.0 kcal mol^–1^. This is most likely related to the significant structural reorganization needed to bring the N–H proton in proximity to the hydride. Under catalytic conditions an excess of HCOOH is present, so we decided to investigate whether an additional equivalent of HCOOH could mediate the proton transfer from the ligand to the Ir–H. Indeed, a transition state was obtained wherein protonation of the Ir–H with HCOO*H* occurred simultaneously with reprotonation of HC*O*OH by the ligand N–H (Fig. 6, green profile). This transition state (**4IV′**) turned out to be 13.7 kcal mol^–1^ lower in energy (10.3 kcal mol^–1^) compared to transition state **4IV**. Similar second-sphere interactions were previously proposed in the heterolysis of H_2_ assisted by exogenous water.^12^ In our case, HCOOH provides a perfect geometrical fit between Ir–H and the N–H moiety of the ligand for second-sphere assisted proton transfer to occur.

### Computational investigation into direct hydride-transfer

**Fig. 7:**
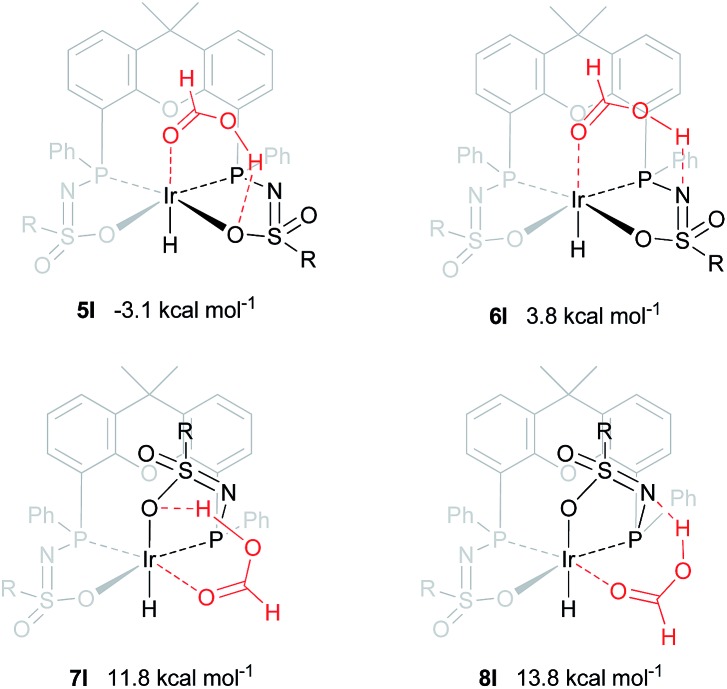
DFT optimized geometries of formic acid adducts **5I–8I** (BP86, def2-TZVP, Δ*G*0298K in kcal mol^–1^), R = phenyl.

An alternative mechanism for the dehydrogenation of formic acid could involve a direct hydride-transfer of the formate hydrogen (*H*COOH) to the Ir-center, subsequent to, or in concert with, proton transfer of the acidic HCOOH proton to the ligand scaffold. In this mechanism a single metal coordination site is sufficient for effective turnover. To test the validity of such a pathway, we computed different HCOOH–**2** adducts. Adducts with either axial or equatorial coordination were all found to exhibit stabilizing hydrogen-bonding interactions with either the nitrogen atom or the coordinated oxygen atom in the ligand scaffold, resulting in structures **5I–8I** (see Fig. 7). Axial coordination of HCOOH (**5I**; interaction with O) is the only structure found to be exergonic, by 3.07 kcal mol^–1^, compared to the free complex plus HCOOH. Formation of structure **6I**, with an N–H interaction, is endergonic by 3.82 kcal mol^–1^. Equatorial coordination of HCOOH, leading to structures **7I** or **8I**, is associated with a significant energy penalty of 11.8 (**7I**) or 13.8 (**8I**) kcal mol^–1^ compared to formation of the axial adducts.

**Fig. 8:**
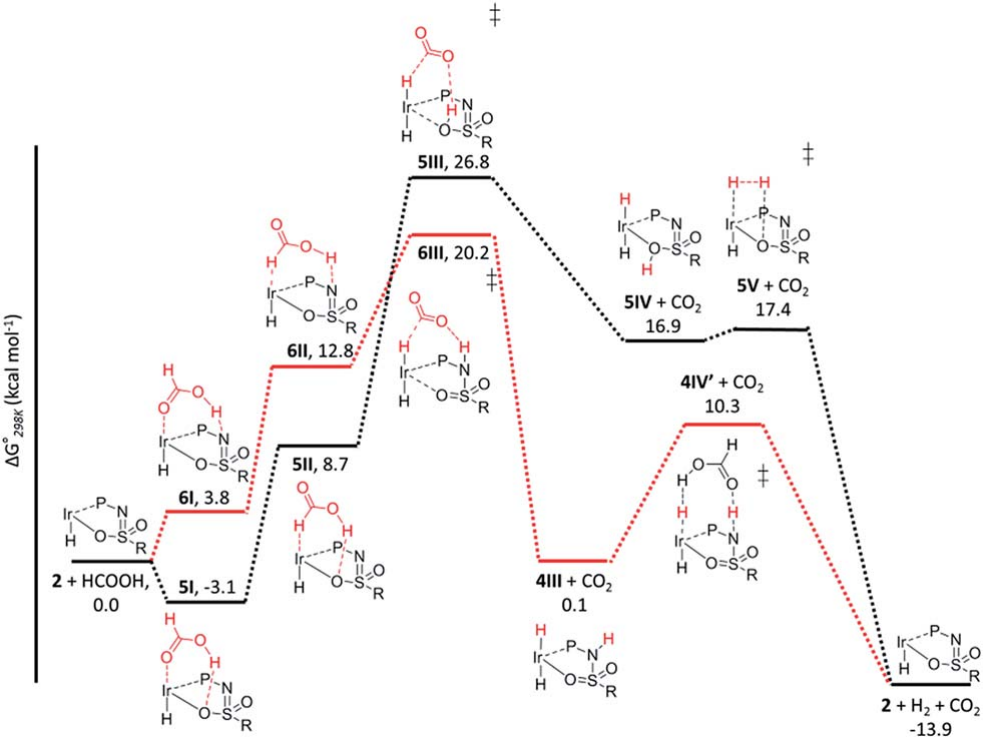
Potential energy diagram (DFT, BP86, def2-TZVP) for dehydrogenation of formic acid by **5I** (black profile) and **6I** (red profile); Δ*G*0298K is in kcal mol^–1^.

**Fig. 9:**
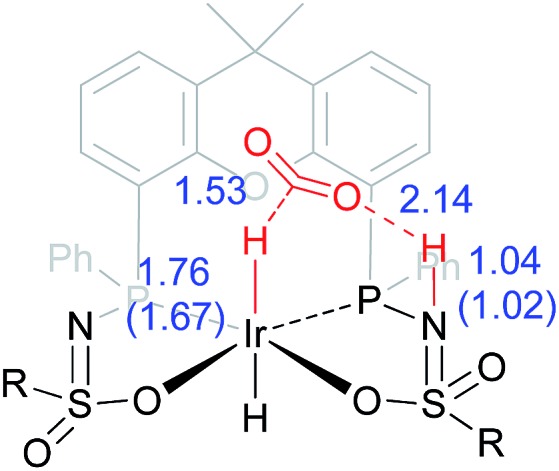
Transition state **6III** with stabilizing hydrogen-bond between ligand and CO_2_. Relevant computed bond lengths (in Å) are compared to values found computationally for structure **4III** (in parentheses).

The dehydrogenation of HCOOH *via* a direct hydride-transfer pathway was first investigated using the axial HCOOH adducts **5I** and **6I**. Starting from the energetically most stable structure **5I** (see black energy profile in Fig. 8), an endergonic rearrangement to **5II** was found (+11.8 kcal mol^–1^), which orients the formate hydrogen in a favorable position for direct hydride-transfer to the metal. In transition state **5III** (Δ*G* ^‡^ = 26.8 kcal mol^–1^ and Δ*G* ^‡^ = 29.9 kcal mol^–1^ with respect to **5I**) HCOOH is fully deprotonated by the ligand. This enables facile expulsion of CO_2_ leading to a 6-membered Ir···H···C···O···H···O transition state. After release of CO_2_, dihydride complex **5IV** is formed in an overall endergonic process (+16.9 kcal mol^–1^), whereafter protonation of the iridium-hydride *via* transition state **5V** (+17.4 kcal mol^–1^) releases H_2_. The HCOOH dehydrogenation pathway was also investigated starting from structure **6I** (red profile in Fig. 8). The rearrangement from **6I** to **6II** (similar to the rotation of **5I** to **5II**) is endergonic by 12.8 kcal mol^–1^. However, transition state **6III** was found to be significantly lower in energy (Δ*G* ^‡^ = 20.2 kcal mol^–1^) compared to **5III** (Δ*G* ^‡^ = 26.8 kcal mol^–1^), leading to formation of structure **4III** *via* an unusual 8-membered Ir···H···C···O···H···N···S···O transition state, see Fig. 6. Structures **6I–III** are all stabilized by hydrogen bonding interactions between HCOOH/CO_2_ and the ligand pre-assembling HCOOH and stabilizing the transition state. The same role of the ligand was found in the energy profile starting from structure **5I**. The Ir–H and N–H bonds in transition state **6III** are elongated compared to those found in **4III** (Ir–H: 1.76 Å *vs.* 1.67 Å; N–H: 1.04 Å *vs.* 1.02 Å), which indicates a late transition state (Fig. 9). A similar interaction was proposed by Hazari *et al.* for CO_2_ insertion of an Ir–H stabilized by hydrogen bonding between a ligand-based N–H group and CO_2_.^13^ Calculations performed on structures lacking these H-bonding interactions (by pointing the N–H fragment outwards) yielded unstable structures and no transition states could be found. After CO_2_ release from **6III**, structure **4III** is formed, which releases H_2_ with the assistance of another molecule of HCOOH (structure **4IV′**), regenerating the catalyst as described above (Fig. 6, green profile). Direct hydride-transfer was also investigated starting from structures **7I** and **8I**, but these energy profiles were found to be significantly higher than for **5I** and **6I**, see ESI.†

**Scheme 3:**
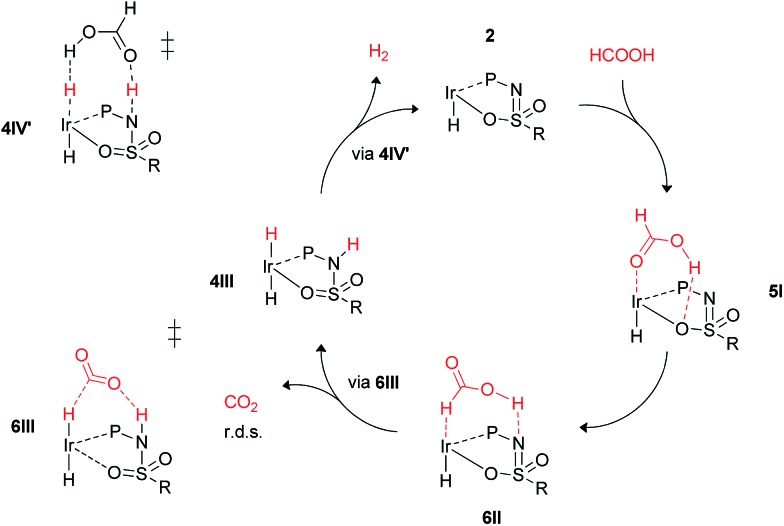
Schematic proposed catalytic cycle of the ligand-assisted dehydrogenation of HCOOH by bisMETAMORPhos complexes **2a–c**.

These calculations suggest that HCOOH dehydrogenation using bisMETAMORPhos-derived complexes **2a–c** follows an outer-sphere direct hydride-transfer mechanism at the axial vacant site of these monohydride species. Starting from formic acid adduct **5I**, the energetically most favored pathway requires rearrangement of **5I** to **6I**. This is likely a facile process due to the high proton-mobility within the ligand scaffold (from N–H to O–H), as was also inferred from the rapid proton-shuttling in complexes **1a–c**. This results in an overall activation barrier of 23.3 kcal mol^–1^, taking **5I** as the resting state. This is in agreement with the variable temperature ^1^H NMR observations, as the change in chemical shift upon addition of *H*COOH to **2a** indicates the formation of a **2a**–HCOOH adduct akin to structure **5I**. Protonation of the ligand takes place in transition state structure **6III**. The hydride ligand already present in complexes **2a–c**, *trans* to the axial coordination site of HCOOH, acts purely as a spectator ligand. The lower activity of complex **2c**, bearing electron-withdrawing CF_3_ groups, can therefore potentially be explained by the decreased basicity of the nitrogen atom in the ligand. The electronic effect was also investigated theoretically by comparing *p*-CF_3_ with *p*-CH_3_ substituents. Indeed, a higher activation barrier was found for *p*-CF_3_ (24 kcal mol^–1^) compared to *p*-CH_3_ (22.5 kcal mol^–1^), see ESI† for energy profiles. We propose the following overall dual role for the bisMETAMORPhos ligand in the mechanism of formic acid dehydrogenation (Scheme 3). Species **2** coordinates HCOOH at the vacant axial site, aided by hydrogen-bonding with the coordinated sulfon–oxygen atom to give structure **5I**. Reorientation of the formate group gives a pre-activated *H*COOH unit that participates in hydrogen-bonding with the deprotonated nitrogen of the ligand arm (**6II**). Release of CO_2_ is achieved *via* transition state **6III** (rate determining step) wherein the ligand deprotonates HCOOH and stabilizes the direct hydride transfer by a hydrogen bonding interaction. This gives *trans*-dihydride **4III** that releases H_2_ aided by an additional equivalent of HCOOH, which protonates the Ir–H and in turn is reprotonated by the ligand, thereby regenerating starting complex **2**. The reversibility of this catalytic system is currently under investigation.

## Conclusions

We report several bisMETAMORPhos ligands (**La–Lc**) based on the xanthene backbone bearing two sulfonamide-phosphine units. The prototropic nature of these PN(H)SO_2_R fragments results in different ratios for the P^III^ and P^V^ tautomers, depending on the electronic and steric nature of the sulfonamide substituents. Formation of Ir^I^ complexes **1a–c** was achieved by coordination of **La–Lc** to Ir(acac)(cod). Subsequent formal oxidative addition of the remaining N–H group resulted in the formation of the corresponding Ir^III^-monohydride complexes Ir^III^(H)(**L**) (**2a–c**). These three complexes are active catalysts for the base-free dehydrogenation of formic acid, with TOFs of 3090, 877 and 1791 h^–1^ for **2a–c**, respectively. These data reflect the influence of subtle electronic and steric changes in the ligand architecture. The role of the ligand during catalysis was investigated by variable temperature ^1^H and ^31^P NMR spectroscopic measurements and DFT calculations. Variable temperature NMR data point to the formation of a **2a**–HCOOH adduct. DFT calculations indicate that the hydrogen-bonding abilities of the ligand play an important role in the mechanism, resulting in an uncommon direct hydride-transfer mechanism instead of the more commonly proposed β-hydride elimination for HCOOH dehydrogenation. This was also found to be the rate-determining step (23.3 kcal mol^–1^), which confirms experimental observations using DCOOH and HCOOD.^4*f*^ A similar mechanism has been proposed for Al- and Fe-based systems reported by Berben and Milstein, respectively.^6c,8c^ It was found that the combined hydrogen-bonding and proton-responsive properties of the bisMETAMORPhos ligands are essential for the reactivity of complexes **2a–c**. These interactions facilitate the pre-assembly of the HCOOH substrate and the stabilization of catalytically relevant intermediates and transition states, allowing an otherwise inaccessible reaction pathway. We thus show that a single coordination site is effective for the dehydrogenation reaction to occur when using a functional ligand scaffold. The proton-responsive and hydrogen-bonding features of ligands (**La–Lc**) are currently being explored for other reactions. The mechanistic findings presented herein potentially play a role in other HCOOH dehydrogenative catalysts bearing hydrogen-bonding functionalities in their ligand systems.
