# Supplementary material for: Dehydrogenation of formic acid by Ir–bisMETAMORPhos complexes: experimental and computational insight into the role of a cooperative ligand
Source: Chem Sci. 2014 Oct 22;6(2):1027–34. doi: 10.1039/c4sc02555e (PMC5811074; doi:10.1039/c4sc02555e)
Supplement: Supplementary file 1 [file SC-006-C4SC02555E-s001.pdf]

Electronic Supplementary Material (ESI) for Chemical Science.
This journal is © The Royal Society of Chemistry 2014

Supporting information for

Dehydrogenation of Formic Acid by Ir-bisMETAMORPhos Complexes: Experimental and Computational Insight in the Role of a Cooperative Ligand

Sander Oldenhof,[a] Martin Lutz,[b] Bas de Bruin,[a] Jarl Ivar van der Vlugt,[a] Joost N. H. Reek*[a]

[a] S. Oldenhof, Prof. Dr. B. de Bruin, Dr. Ir. J. I. van der Vlugt, Prof. Dr. J. N. H. Reek, van 't Hoff Institute for Molecular Sciences, University of Amsterdam, Science Park 904, 1098 XH, Amsterdam (The Netherlands), E-mail: j.n.h.reek@uva.nl
[b] Dr. M. Lutz, Bijvoet Center for Biomolecular Research Utrecht University¸ Padualaan 8, 3584 CH Utrecht (The Netherlands).

Table of content

General information **S3**

Syntheses, charaterization **S4**

VT-NMR of diastereo-pure complex 2a **S10**

VT-NMR of diastereo-pure complex 2a with 1eq. HCOOH **S11**

Crystal structures of 2c **S12**

Diastereomeric structure used in calculations **S14**

Energies and imaginairy frequencies of calculated structures **S14**

Energy profiles structures 3I and 4I **S15**

Energy profiles structures 5I and 6I **S16**

Energy profile structure 7I **S17**

Energy profiles with CF_3 and CH_3 sustituents **S18**

NMR spectra **S19**

References **S36**

General information

General procedures: All reactions were carried out in dry glassware under nitrogen atmosphere using standard Schlenk techniques unless stated otherwise. THF, dioxane, toluene, pentane were distilled from sodium under dinitrogen, CH_2Cl_2 and diethylether were collected from an MB SPS-800. Deuterated solvents were degassed by four freeze-pump-thaw cycles and dried over molecular sieves (4Å). NMR spectra were measured on a Bruker AMX 400 (^{1}H: 400.1 MHz, ^{13}C: 100.6 MHz and ^{31}P: 162.0 MHz) or on a Varian Mercury 300 (^{1}H: 300.1 MHz) spectrometer at 298 K unless noted otherwise. High resolution mass spectra were recorded on a JEOL JMS SX/SX102A four sector mass spectrometer; for FAB-MS 3-nitrobenzyl alcohol was used as matrix. ESI (electrospray ionization) mass spectra were obtained on a time-of-flight JEOL AccuTOF LC-plus mass spectrometer (JMS-T100LP) equipped with a CSI or ESI source. Calculated spectra were obtained with JEOL Isotopic Simulator (version 1.3.0.0).

Materials: All reagents were purchased from commercial suppliers and used without further purification: Dichlorophenylphosphine (Sigma Aldrich), diethylamine (Sigma Aldrich), 9,9-dimethyl-xanthene (Sigma Aldrich), TMEDA (Sigma Aldrich), nBuLi (Acros organics), phosphorus trichloride (Sigma Aldrich), 4-butylbenzene-1-sulfonamide (ABCR GmbH), 4-(trifluoromethyl)benzenesulfonamide (ABCR GmbH), 2,4,6-tris(isopropyl)benzenesulfonamide (ABCR GmbH), Ir(acac)(COD) (Strem Chemicals), formic acid (Acros organics).

Catalytic dehydrogenation experiments

Catalyst **2a**, **2b** or **2c** (5.0 μmol) was added to toluene (1 mL) in a Schlenk equipped with a condenser and connected to a water replacement set-up.[S1] The reaction mixture was heated to the required temperature and stirred for 10 minutes. Formic acid was added to the reaction mixture (188.6 μL, 5 mmol) and the evolved gas was collected.

The set-up was calibrated with a Brooks flow-meter type 1054-3C and evolved gases were analyzed with a G·A·S Compact GC (Rt-MSieve 5A 20 m × 0.32 mm + Rt-Q-bond 2 m × 0.32 mm). The amounts of mol converted were determined from the volumes of gas collected using equation 1a and 1b.

Determination of molecular volume of H_2 and CO_2

$$V_{H2} = \frac{RT}{p} + b - \frac{a}{RT} = 24.49\ \frac{L}{mol} \qquad \text{1a}$$

R: 8.3145 m^3 Pa•mol^{-1}•K^{-1}

T: 298.15 K

p: 101325 Pa

b: 26.7 • 10^{-6} m^3•mol^{-1}

a: 2.49 • 10^{-10} Pa•m^3•mol^{-2}

$$V_{CO2} = \frac{RT}{p} + b - \frac{a}{RT} = 24.42\ \frac{L}{mol} \qquad \text{1b}$$

R: 8.3145 m^3 Pa•mol^{-1}•K^{-1}

T: 298.15 K

p: 101325 Pa

b: 42.7 • 10^{-6} m^3•mol^{-1}

a: 36.5 • 10^{-10} Pa•m^3•mol^{-2}

Computational details:

Geometry optimizations were carried out with the Turbomole program package[S2], coupled to the PQS Baker optimizer[S3] via the BOpt package[S4], at the spin unrestricted ri-DFT level using the BP86 functional[S5], the resolution-of-identity (ri) method[S6], and the def2-TZVP basis set[S7] for the geometry optimizations. Energy profiles are shown below and all structure are conveniently added as separate .xyz and .pdb files.

Synthesis and characterization.

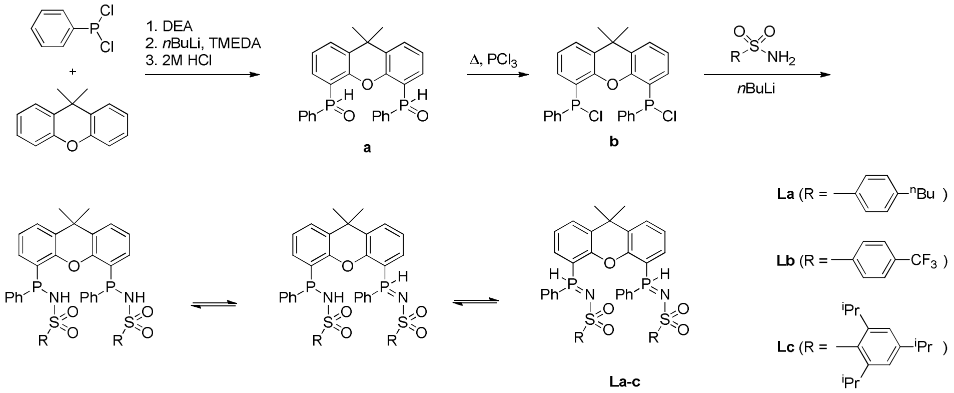

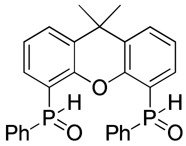

a

(9,10-dihydroanthracene-1,8-diyl)bis(phenylphosphine oxide) (a)

To a solution of dichlorophenylphosphine (6.98 g, 5.29 mL, 39.0 mmol) in Et_2O (150 mL) at 0 °C was added diethylamine (5.73 g, 8.08 mL, 78.38 mmol) dropwise under vigorous stirring. A white precipitate formed while the reaction mixture was allowed to warm up to room temperature and stirred for 16 hours. The reaction mixture was filtered, concentrated and *N*,*N*-(diethylamino)chlorophenylphosphine was obtained as a yellow oil, which was used immediately for follow-up synthesis due to its instability. **^{31}P{^{1}H}** NMR (162.0 MHz, Et_2O unlocked): δ = 138.96.

To a solution of 9,9-dimethylxanthene (4.0 g, 19.02 mmol) and TMEDA (4.53 g, 5.85 mL, 39.0 mmol) in diethylether (150 mL) was added a solution of nBuLi (15.3 mL, 2.5 M in hexane, 38.24 mmol) at 0 °C and a deep purple/brown solution was obtained. The reaction mixture was allowed to warm up to room temperature and stirred overnight. *N*,*N*-(diethylamino)-chlorophenylphosphine (39.0 mmol) in diethylether (75 mL) was added dropwise to the reaction mixture and a clear yellow suspension was obtained and stirred for 16 hours. **^{31}P{^{1}H}** NMR (162.0 MHz, Et_2O unlocked): δ = 52.55 (s), 51.74 (s), [racemic mixture of diastereomers (*RR*/*SS*, *SR*/*RS*) of the phosphinamine]. The reaction mixture was carefully quenched with a 2 M HCl solution (100 mL) and stirred for 1 hour. The phases were separated and the aqueous phase was extracted with ethylacetate (3×). The organic phases were combined and concentrated. Azeotropic drying with toluene (2×) and stripping with

diethylether (3×) yielded a white foam. Purification by column chromatograpy (SiO_2/H_2O 8:2, eluens Et_2O/MeOH 97:3, deposited in CH_2Cl_2) yielded **a** as a racemic mixture of diastereomers (*RR*/*SS*, *RS*/*SR*) as a white foam (4.11g, 47% yield).

^{31}P{^{1}H} NMR (162 MHz, $CDCl_3$): δ = 11.76 (s), 11.28 (s);

^{31}P NMR (162 MHz, $CDCl_3$): δ = 11.76 (dq, *J* = 506.8, 14.2 Hz), 11.28 (dq, *J* = 506.2, 14.1 Hz);

^{1}H NMR (400 MHz, CD_2Cl_2): δ = 8.22 (d, *J* = 506.8, 4H, P<u>H</u>), 8.16 (d, *J* = 506.2, 4H, P<u>H</u>), 7.83-7.45 (m, 56H), 7.30 (m, 8H), 1.76 (s, 6H, CH_3-Xanthene), 1.74 (s, 12H, CH_3-Xanthene), 1.67 (s, 6H, CH_3-Xanthene);

^{13}C{^{1}H} NMR (100 MHz, CD_2Cl_2): δ = 150.90 (d, *J* = 3.11 Hz), 150.78 (d, *J* = 3.16 Hz), 132.80 (d, *J* = 2.86 Hz), 132.68 (d, *J* = 2.90 Hz), 132.12 (d, *J* = 25.62 Hz), 131.83 (d, *J* = 2.14 Hz), 131.76 (d, overlapping), 131.73 (d, overlapping), 131.66 (d, *J* = 1.85), 331.11 (s), 131.09 (d, *J* = 25.93 Hz), 131.04 (s), 130.61 (s), 130.55 (s), 129.24 (d, *J* = 6.47 Hz), 129.11 (d, *J* = 6.47 Hz), 124.64 (d, *J* = 11.01 Hz), 124.52 (d, *J* = 11.01 Hz), 120.0 (d, *J* = 23.56 Hz), 119.04 (d, *J* = 23.56 Hz), 34.46 (s), 33.71 (s), 32.90 (s), 31.98 (s);

HR MS (FAB^+): *m*/*z* calcd. for $C_{27}H_{25}O_3P_2$ $[M+H]^+$: 459.1279, observed: 459.1275.

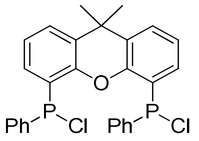

b

(9,9-dimethyl-9H-xanthene-4,5-diyl)bis(chloro(phenyl)phosphine) (b)

Compound **a** (1.39 g, 3.03 mmol) was dissolved in neat PCl_3 (5 mL) at 0 °C and heated to 60 °C for 14 hours, during which time a yellow/orange suspension was obtained. The reaction mixture was cooled to room temperature, concentrated, dissolved in 10 mL toluene and evaporated (3×) to leave a yellow foam. Compound **b** (stereo-isomers *RR*, *SS*, *RS*, *SR*) is unstable and should be used immediately.

^{31}P{^{1}H} NMR (162.0 MHz, THF unlocked): δ = 73.71 (s), 73.65 (s).

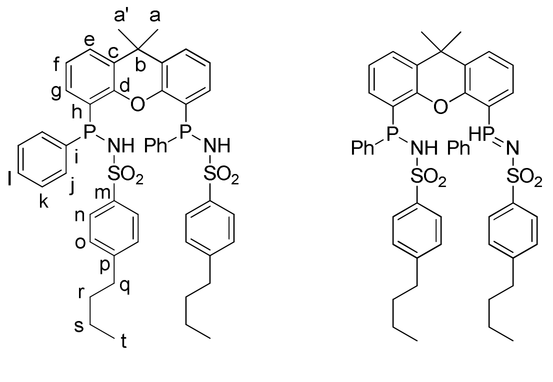

	**La1**	**La2**
**Ratio:**	**1**	**0.4**

***N*,*N*'-((9,9-dimethyl-9H-xanthene-4,5-diyl)bis(phenylphosphinediyl))bis(4-butylbenzenesulfonamide) (La)**

Commercially available 4-butylbenzene-1-sulfonamide (1.30 g, 6.08 mmol) was dissolved in 10 mL of toluene and azeotropically dried. The compound was dissolved in THF (25 mL) and nBuLi (2.55 mL, 2.5 M in hexane, 6.36 mmol) was added dropwise at 0 °C, resulting in a white/grey slurry. Compound **b** (3.04 mmol) was dissolved in THF (30 mL) and slowly added to the slurry to give a clear yellow solution that was stirred at room temperature for 14 hours. The reaction mixture was concentrated and purified by column chromatograpy (SiO_2, eluens toluene/ethyl acetate 9:1, deposited in CH_2Cl_2). Fractions

were combined and concentrated, stripping with Et_2O (3×) yielded **La** as a white foam (0.9 g, 1.06 mmol, 35% yield). Compound **La** was obtained pure in its mesomeric form (*RS*/*SR*) and this species exists in two tautomeric forms **La1** and **La2**, with a ratio of 1 : 0.4, respectively, according to ^{1}H and ^{31}P NMR integrations.

$^{31}P\{^{1}H\}$ NMR (162 MHz, CD_2Cl_2): δ = 26.92 (s, **La1**), 23.24 (d, *J* = 39.1 Hz, **L1b**), -6.13 (d, *J* = 39.1 Hz, **La2**);
^{31}P NMR (162 MHz, CD_2Cl_2): δ = 26.92 (s), 23.24 (d, *J* = 39.1 Hz), -6.13 (dd, *J* = 518.2, 39.1 Hz);
^{1}H NMR: (400 MHz, CD_2Cl_2): Major tautomer **La1**: δ = 7.76 (dt, *J* = 7.23, 1.09 Hz, 2H), 7.49 (br m, 4H), 7.37 (br m, 2H), 7.21 (br m, 8H), 5.96 (br s, 2H, N<u>H</u>), 2.56 (t, *J* = 7.93 Hz, 4H, (C_3H_7)-C<u>H</u>$_2$-Ar), 1.57 (s, 3H, C<u>H</u>$_3$-Xanthene), 1.56-1.49 (m, 4H, (C_2H_5)-C<u>H</u>$_2$-CH_2-Ar), 1.47 (s, 3H, C<u>H</u>$_3$-Xanthene), 1.38-1.26 (m, 4H, CH_3-C<u>H</u>$_2$-(C_2H_4)-Ar), 0.91 (t, *J* = 7.3 Hz, 6H, C<u>H</u>$_3$-(C_3H_6)-Ar) Minor tautomer **La2**δ = 8.68 (dd, *J* = 518.4, 5.6 Hz, 0.4H, P<u>H</u>) remaining signals are overlapped by tautomer **La1**;
$^{13}C\{^{1}H\}$ NMR (101 MHz, CD_2Cl_2): δ = 151.57 (d, *J* = 16.8 Hz, d, **La1**), 148.08 (s, m, **La1**), 138.91 (s, p, **La1**), 130.77 (s), 130.56 (s), 130.16 (s), 130.06 (d, *J* = 8.55 Hz), 129.05 (s), 128.73 (s), 128.35 (s), 128.30 (s), 126.63 (s), 123.80 (s), 122.74 (d, *J* = 18.0 Hz, h/i, **La1**), 35.43 (s, q, **La1**), 34.22 (s, b, **La1**), 33.33 (s, a/a',**La1**), 33.14 (s, r, **La1**), 32.61 (s, a/a',**La1**), 22.25 (s, s, **La1**), 13.63 (s, t, **La1**);
HR MS (FAB^+): *m*/*z* calcd. for $C_{47}H_{51}N_2O_5P_2S_2$ $[M+H]^+$: 849.2715, observed: 849.2662;
Anal. Calcd. for $C_{47}H_{50}N_2O_5P_2S_2$: C, 66.49; H, 5.94; N, 3.30, found: C, 66.27; H, 5.99, N, 3.29.

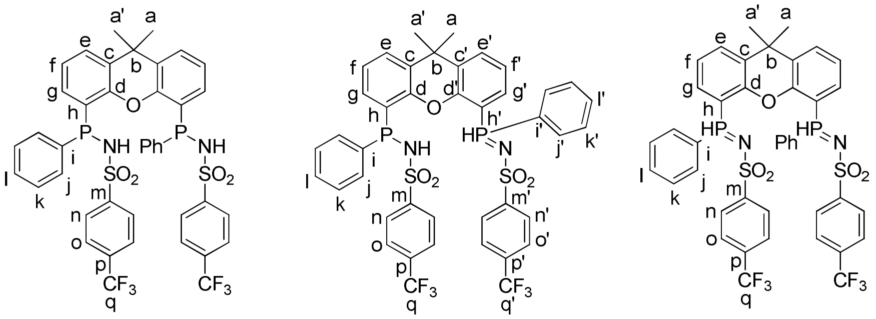

Lb1 **Lb2** **Lb3**

Ratio: **1** **1.8** **0.2**

***N,N'*-((9,9-dimethyl-9H-xanthene-4,5-diyl)bis(phenylphosphinediyl))bis(4-trifluoromethylbenzenesulfonamide) (Lb)**
Commercially available 4-(trifluoromethyl)benzenesulfonamide (0.459 g, 2.04 mmol) was dissolved in 4 mL of toluene and azeotropically dried. The compound was dissolved in THF (15 mL) and nBuLi (0.86 mL, 2.5 M in hexane, 2.14 mmol) was added dropwise at 0 °C, resulting in a white slurry. Compound **b** (1.02 mmol) was dissolved in THF (10 mL) and slowly added to the slurry to give a clear yellow solution that was stirred at room temperature for 16 hours. The reaction mixture was concentrated and purified by column chromatograpy (SiO_2, eluens toluene/ethyl acetate 9:1, deposited in CH_2Cl_2). Fractions were combined and concentrated, stripping with Et_2O (3×) yielded **Lb** as a white foam (0.258 g, 0.3 mmol, 29% yield). Compound **Lb** was obtained pure in its mesomeric form (*RS*/*SR*) and this species exists in two tautomeric forms **Lb1, Lb2 and Lb3** with a ratio of 1 : 1.8 : 0.2, respectively, according to ^{1}H and ^{31}P NMR integrations.

$^{31}P\{H\}$ NMR (162 MHz, CD_2Cl_2) δ 27.07 (s, **Lb1**), 24.07 (d, *J* = 40.7 Hz, **Lb2**), -4.10 (s, **Lb3**), -6.07 (d, *J* = 40.7 Hz, **Lb2**);

^{31}P NMR (162 MHz, CD_2Cl_2) δ 27.07 (s), 24.07 (d, *J* = 40.7 Hz), -4.10 (d, *J* = 526.5 Hz), -6.07 (d, *J* = 520.7 Hz);
^{1}H NMR: (400 MHz, CD_2Cl_2): Major tautomer **Lb2**: δ = 8.76 (dd, *J* = 520.5, 5.8 Hz, 1H, P<u>H</u>) 7.98 – 7.92 (m, 2H), 7.73 (d, *J* = 8.2 Hz, 2H), 7.62 (d, *J* = 8.3 Hz, 2H), 7.55 (d, *J* = 8.2 Hz, 2H), 7.49 (d, *J* = 8.1 Hz, 2H), 7.38 – 7.33 (m, 2H), 7.30 – 7.17 (m, 10H), 7.09 (t, *J* = 7.4 Hz, 2H), 6.33 (d, *J* = 4.4 Hz, 1H, N<u>H</u>), 1.67 (s, 3H, C$\underline{H}_3$-Xanthene), 1.47 (s, 3H, C$\underline{H}_3$-Xanthene); Tautomer **Lb1** δ = 6.21 (s, 1H, N<u>H</u>), 1.50 (s, 3H, C$\underline{H}_3$-Xanthene), 1.40 (s, 3H, C$\underline{H}_3$-Xanthene), remaining signals are overlapped by tautomer **Lb2 /3**;
Tautomer **Lb3** δ = 8.80 (s, 2H, P<u>H</u>) remaining signals are overlapped by tautomer **Lb1/2**;
^{13}C{^{1}H} NMR (101 MHz, CD_2Cl_2): δ = 152.08 (d, *J* = 16 Hz), 151.89 – 151.77 (m), 151.59 (d, *J* = 20.9 Hz), 149.18 (s), 145.54 (s), 145.43 (s), 136.24 – 136.04 (m), 135.90 (d, *J* = 10.0 Hz), 134.37 (s), 134.01 (d, *J* = 7.5 Hz), 133.66 (s), 133.63 (s), 133.52 (s), 133.31 (s), 132.45 (s), 132.38 (s), 132.33 (s), 132.15 (s), 132.10 (s), 131.80 (s), 131.68 (s), 131.39 (s), 131.21 (s), 131.19 (s), 131.01 (s), 130.83 (s, c/c‘, **Lb1/ Lb2**), 130.50 (s, c/c‘, **Lb1/ Lb2**), 130.46 (s, c/c‘, **Lb1/ Lb2**), 130.27 (s), 130.12 (s), 129.93 (s), 129.83 (s), 129.79 (s), 129.53 (s), 129.46 (s), 129.32 (s), 129.13 (s), 129.07 (s), 128.92 (s), 128.78 (s), 128.73 (s), 127.76 (d, *J* = 13.5 Hz), 127.62 (s), 126.82 (s), 126.68 - 126.46 (m), 126.41 – 126.18 (m), 126.04 – 125.79 (m), 125.35 (s), 125.07 (s), 124.95 (s), 124.47 (s), 124.35 (m, i‘/h‘, **Lb1**), 123.28 (d, *J* = 18.3 Hz, i‘/h‘,**Lb2**), 122.47 (m, q/q‘,**Lb2** / **Lb3**), 113.03 (s, i/h, **Lb2**), 111.97 (s, i/h, **Lb2**), 34.85 (s, b, **Lb2**), 34.66 (s, b, **Lb1**), 34.53 (s, b, **Lb3**), 33.68 (s, a/a‘, **Lb2**), 33.42 (s, a/a‘, **Lb3**), 32.65 (s, a/a‘, **Lb1**), 32.01 (s, a/a‘, **Lb3**), 29.88 (s, a/a‘, **Lb1**), 29.56 (s, a/a‘,**Lb2**).
^{19}F NMR (282 MHz, CD_2Cl_2) δ -63.25 (m);
HR MS (ESI$^+$): *m/z* calcd. for $C_{57}H_{70}$ $N_2O_5P_2S_2$ $[M+H]^+$: 873.1210, observed: 873.1234;
Anal. Calcd. for $C_{41}H_{32}F_6N_2O_5P_2S_2$: C, 56.42; H, 3.70; N, 3.21, found: C, 56.62; H, 3.72, N, 3.17.

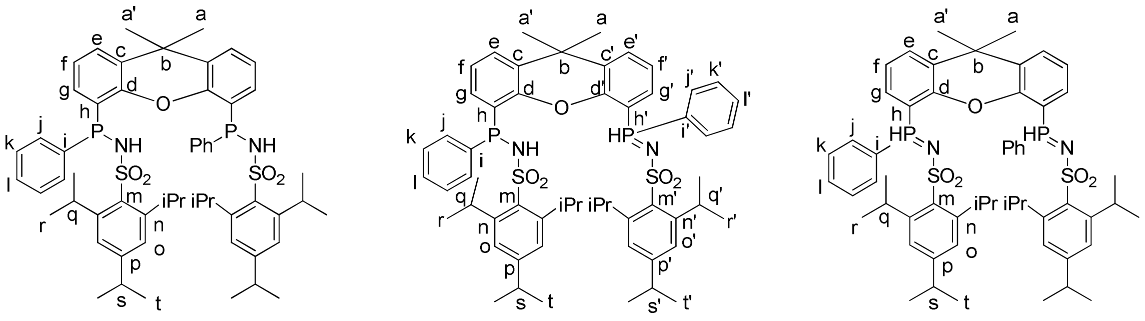

	Lc1	Lc2	Lc3
Ratio:	1	1.9	0.1

***N,N'*-((9,9-dimethyl-9H-xanthene-4,5-diyl)bis(phenylphosphinediyl))bis(2,4,6-triisopropylbenzenesulfonamide) (Lc)**
Commercially available 2,4,6-triisopropylbenzenesulfonamide (1.73 g, 6.10 mmol) was dissolved in 8 mL of toluene and azeotropically dried. The compound was dissolved in THF (25 mL) and nBuLi (2.56 mL, 2.5 M in hexane, 6.4 mmol) was added dropwise at 0 °C, resulting in a white slurry. Compound **b** (3.05 mmol) was dissolved in THF (20 mL) and slowly added to the slurry to give a clear yellow solution that was stirred at room temperature for 16 hours. The reaction mixture was concentrated and purified by column chromatograpy (SiO_2, eluens toluene/ethyl acetate 100:2 to 100:6, deposited in CH_2Cl_2). Fractions were combined and concentrated, stripping with Et_2O (3×) yielded **Lc** as a white foam (1.15 g, 1.16 mmol, 38% yield). Compound **Lc** was obtained pure in its mesomeric form (*RS*/*SR*) and this species exists in two tautomeric forms **Lc1, Lc2 and Lc3** with a ratio of 1 : 1.9 : 0.1, respectively, according to ^{1}H and ^{31}P NMR integrations.

^{31}P{H} NMR (162 MHz, CD_2Cl_2) δ 23.89 (s, **Lc1**), 21.81 (d, *J* = 31.0 Hz, **Lc2**), -7.76 (s, **Lc3**), -10.10 (d, *J* = 31.0 Hz, **Lc2**).
^{31}P NMR (162 MHz, CD_2Cl_2) δ 23.89 (s), 21.81 (br. d, *J* = 31.0 Hz), -10.10 (br. dd, *J* = 521.8 31.0 Hz).
^{1}H NMR: (400 MHz, CD_2Cl_2): Major tautomer **Lc2**: δ = 8.57 (dd, *J* = 519.0, 4.1 Hz, 1H, PH), 7.68 – 7.39 (m, 4H), 7.36 – 7.21 (m, 2H), 7.17 (s, 1H), 7.13 (s, 1H), 7.06 (s, 1H), 7.22 – 6.80 (m, 11H), 5.41 (d, *J* = 5.4 Hz, 1H, NH), 4.54 – 4.37 (m, 2H, $(CH_3)_2$-CH-Ar (para)), 4.18 – 3.98 (m, 2H, $(CH_3)_2$-CH-Ar (NH-ortho)), 3.01 – 2.79 (m, 2H, $(CH_3)_2$-CH-Ar (PH-ortho)), 1.74 (s, 3H, CH_3-Xanthene), 1.54 (s, 3H, CH_3-Xanthene), 1.29 – 1.23 (m, 24H, $(CH_3)_2$-CH-Ar (ortho)), 1.09 (d, *J* = 6.8, 6H, $(CH_3)_2$-CH-Ar (PH-para)), 1.08 (d, *J* = 6.8, 6H, $(CH_3)_2$-CH-Ar (NH-para));
Tautomer **Lc1** δ = 5.53 (br s, 2H, NH), 1.69 (s, 3H, CH_3-Xanthene), 1.52 (s, 3H, CH_3-Xanthene), 1.19 (d, *J* = 6.8 Hz, 12H, $(CH_3)_2$-CH-Ar (ortho)), 1.06 (d, *J* = 6.7 Hz, 6H, $(CH_3)_2$-CH-Ar (para)) remaining signals are overlapped by tautomer **Lc2/3**;
Tautomer **Lc3** δ = 8.83 (s, 2H, PH) remaining signals are overlapped by tautomer **Lc1/2**;
^{13}C{^{1}H} NMR (75 MHz, CD_2Cl_2): δ = 153.85 (d, *J* = 20.3 Hz), 151.52 (s), 150.65 (s), 149.07 (s), 140.51 (s), 138.59 (s), 138.15 (d, *J* = 11.6 Hz), 136.02 (d, *J* = 18.4 Hz), 133.33 (s), 133.29 (s), 132.94 (s), 132.87 (s), 132.55 (s), 132.07 (s), 131.95 (s), 131.90 (s), 131.71 (s), 131.68 (s), 131.48 (s), 131.24 (s), 130.99 (s), 130.95 (s), 130.86 (s), 130.64 (s), 129.88 (s), 129.64 (s), 129.56 (s), 129.50 (s), 129.32 (s), 129.27 – 128.99 (m), 128.91 (s), 127.13 (s), 125.64 (s), 125.12 (s), 124.96 (s), 124.89 (s), 124.69 (s, o/o‘, **Lc1/2**), 124.59 (s), 124.52 (s, o/o‘, **Lc1/2**), 123.63 (s, o/o‘, **Lc1/2**), 114.38 (s), 113.02 (s). 34.50 (br. s, b, **Lc1/2**), 34.42 (s, a/a‘, **Lc1/2**), 30.41 (s, a/a‘, **Lc1/2**), 30.18 (s, a/a‘, **Lc1/2**), 29.34 (s, a/a‘, **Lc1/2**), 25.01 (s, q/q‘/r/r‘/t/t‘, **Lc1/2**), 24.89 (s, q/q‘/r/r‘/t/t‘, **Lc1/2**), 24.83 (s, q/q‘/r/r‘/t/t‘, **Lc1/2**), 24.64 (s, q/q‘/r/r‘/t/t‘, **Lc1/2**), 24.58 (s, q/q‘/r/r‘/t/t‘, **Lc1/2**), 24.48 (s, q/q‘/r/r‘/t/t‘, **Lc1/2**), 23.78 (s, q/q‘/r/r‘/t/t‘, **Lc1/2**), 23.74 (s, q/q‘/r/r‘/t/t‘, **Lc1/2**), 23.58 (s, q/q‘/r/r‘/t/t‘, **Lc1/2**), 23.55 (s, q/q‘/r/r‘/t/t‘, **Lc1/2**);
HR MS (ESI$^+$): *m/z* calcd. for $C_{57}H_{70}$ $N_2O_5P_2S_2$ $[M+H]^+$: 989.4280, observed: 989.4367; $[M+Na]^+$: 1011.4099, observed: 1011.4174;
Anal. Calcd. for $C_{57}H_{70}N_2O_5P_2S_2$: C, 69.20; H, 7.13; N, 2.83, found: C, 69.14; H, 7.16, N, 2.81.

Complex 1a

Commercially available Ir(acac)(COD) (6 mg, 0.015 mmol) was dissolved in CH_2Cl_2 (1 mL) together with ligand **La** (12.8 mg, 0.015 mmol). The reaction mixture turned bright orange instantly and was stirred for 15 minutes. Evaporation of solvent and volatiles left an orange solid in near-quantitative yield.
^{31}P{^{1}H} NMR (162 MHz, CD_2Cl_2): δ = 31.39 (s);
^{1}H NMR: (400 MHz, CD_2Cl_2): δ = 13.25 (br. s, 1H), 7.83 (m, 2H), 7.76 (br. s, 3H), 7.51 (br. s, 6H), 7.42 (d, *J* = 7.3 Hz, 4Hz), 7.37 (d, *J* = 8.0 Hz, 2H), 7.16 (t, *J* = 7.8 Hz, 3H), 6.81 (d, *J* = 7.5 Hz, 4H), 2.53 (t, *J* = 7.9 Hz, 4H, (C_3H_7)-CH_2-Ar), 1.79 (s, 3H, CH_3-Xanthene), 1.64-1.52 (m, 4H, (C_2H_5)-CH_2-CH_2-Ar), 1.51 (s, 3H, CH_3-Xanthene), 1.34 (q, *J* = 7.3 Hz, CH_3-CH_2-(C_2H_4)-Ar, 4H), 0.93 (t, *J* = 7.4 Hz, 6H, CH_3-(C_3H_6)-Ar).

Complex 1b

Commercially available Ir(acac)(COD) (6 mg, 0.015 mmol) was dissolved in CH_2Cl_2 (1 mL) together with ligand **Lb** (13.1 mg, 0.015 mmol). The reaction mixture turned bright orange instantly and was stirred for 15 minutes. Evaporation of solvent and volatiles left an orange solid in near-quantitative yield.
^{31}P{^{1}H} NMR (162 MHz, CD_2Cl_2): δ = 33.08 (s);

^{1}**H NMR**: (400 MHz, CD_2Cl_2): δ = 13.53 (bs. s, 1H), 7.83 (ddd, *J* = 21.3, 11.3, 7.5 Hz, 3H), 7.68 (d, *J* = 8.1 Hz, 3H), 7.52 (m, *J* = 15.4 Hz, 7H), 7.39 (dd, *J* = 7.6, 1.2 Hz, 3H), 7.26 (m, *J* = 8.3 Hz, 5H), 7.17 (dd, *J* = 14.3, 6.6 Hz, 3H), 1.83 (s, 3H), 1.56 (s, 3H).

Complex 1c

Commercially available Ir(acac)(COD) (6 mg, 0.015 mmol) was dissolved in CH_2Cl_2 (1 mL) together with ligand **Lc** (14.8 mg, 0.015 mmol). The reaction mixture turned bright orange instantly and was stirred for 15 minutes. Evaporation of solvent and volatiles left an orange solid in near-quantitative yield.

^{31}**P{^{1}H} NMR** (162 MHz, CD_2Cl_2): δ = 36.04;

^{1}**H NMR**: (400 MHz, CD_2Cl_2): δ = 11.14 (bs. s, 1H), 7.76 - 766 (m, 4H), 7.46 – 7.27 (m, 6H), 7.17 – 7.07 (m, 4H), 6.90 (s, 4H), 4.44 – 4.37 (m, 2H), 3.94 – 3.85 (m, 4H), 1.89 (s, 3H), 1.50 (s, 3H), 1.22 (dd, *J* = 6.9, 1.8 Hz, 12H), 0.93 (d, *J* = 6.7 Hz, 12H), 0.70 (d, *J* = 6.6 Hz, 12H).

Complex 2a[1]

Complex **1a** was stirred at room temperature in CH_2Cl_2 (1 mL) for 30 hours, during which time a color change from orange to bright yellow, reaction mixture was concentrated. Complex **2a** was formed quantitatively as a diastereomeric mixture.

^{31}**P{^{1}H} NMR** (162 MHz, CD_2Cl_2, offset @ -10 ppm, with ratios): δ = 27.20 (d, *J* = 19.7 Hz, 1.0), 26.18 (d, *J* = 20.6 Hz, 0.2), 14.49 (d, *J* = 19.8 Hz, 1.0), 14.25 (d, *J* = 7.4 Hz, 0.45), 13.35 (d, *J* = 20.0 Hz, 0.2), 9.16 (d, *J* = 7.5 Hz, 0.45), 7.43 (d, *J* = 20.3 Hz, 0.4), 6.57 (d, *J* = 20.4 Hz, 0.8), 1.78 (d, *J* = 21.3 Hz, 0.4), 0.12 (d, *J* = 21.3 Hz, 0.8);

^{1}**H NMR**: (400 MHz, CD_2Cl_2, hydride region with ratios): δ 8.01 – 7.93 (m), 7.88 – 7.79 (m), 7.74 – 7.63 (m), 7.59 – 7.42 (m), 7.40 – 7.21 (m), 7.20 – 7.11 (m), 7.11 – 7.06 (m), 7.06 – 6.95 (m), 6.95 – 6.84 (m), 6.84 – 6.74 (m), 6.65 (d, *J* = 9.2 Hz, 1H), 2.65 – 2.51 (m), 2.14 (s), 2.11 (s), 2.08 (s), 2.03 (s), 1.92 (s), 1.74 (s), 1.59 – 1.49 (m), 1.50 (s), 1.39 – 1.23 (m), 0.99 – 0.93 (m), 0.93 – 0.86 (m), -22.65 (t, *J* = 21.0 Hz, 0.2), -22.74 (t, *J* = 21.7 Hz, 1.0), -24.76 (t, *J* = 25.1 Hz, 0.8) -24.99 (t, *J* = 25.7 Hz, 0.8), -28.66 (t, *J* = 22.0 Hz, 1H, 0.45); **HR MS** (FAB^{+}): *m/z* calcd. for $C_{47}H_{50}IrN_2O_5P_2S_2$ $[M+H]^{+}$: 1041.2266, observed: 1041.2256; **Anal. Calcd.** for $C_{47}H_{49}IrN_2O_5P_2S_2$: C, 54.27; H, 4.75; N, 2.69, found: C, 54.05; H, 4.89, N, 2.73.

Complex 2b

Complex **1b** was stirred at room temperature in toluene (1 mL) for 40 hours at 70 °C, during which time a color change from orange to yellow, reaction mixture was concentrated. Complex **2b** was formed quantitatively as a diastereomeric mixture.

^{31}**P{^{1}H} NMR** (162 MHz, CD_2Cl_2, offset @ -10 ppm, with ratios): δ = 28.70 (d, *J* = 19.8 Hz, 1.0), 27.73 (d, *J* = 20.5 Hz, 0.2), 15.42 (d, *J* = 17.4 Hz, 0.1), 14.95 (d, *J* = 19.8 Hz, 1.0), 13.61 (d, *J* = 20.3 Hz, 0.2), 9.96 (d, *J* = 16.7 Hz, 0.1);

^{1}**H NMR**: (400 MHz, CD_2Cl_2, hydride region with ratios): δ 8.29 (d, *J* = 8.2 Hz), 8.24 (d, *J* = 8.1 Hz), 7.82 (dt, *J* = 16.6, 8.3 Hz), 7.70 (d, *J* = 7.8 Hz), 7.58 (t, *J* = 7.6 Hz), 7.55 – 7.35 (m), 7.33 – 7.19 (m), 7.19 – 7.06 (m), 7.06 – 6.98 (m), 6.98 – 6.87 (m), 6.84 – 6.77 (m), 6.63 – 6.53 (m), 1.92 (s), 1.54 (s), -22.56 (t, *J* = 21.5 Hz, 0.2), -22.64 (t, *J* = 22.0 Hz, 1.0), -28.76 (t, *J* = 22.1 Hz, 0.1);^{19}**F NMR** (282 MHz, CD_2Cl_2) δ -63.22 (s), -63.52 (s); **HR MS** (CSI^{+}): *m/z* calcd. for $C_{41}H_{31}F_6IrN_2O_5P_2S_2$ $[M+H]^{+}$: 1065.0761, observed: 1065.0805; **Anal. Calcd.** for $C_{41}H_{31}F_6IrN_2O_5P_2S_2$: C, 46.28; H, 2.94; N, 2.63, found: C, 46.01; H, 3.04, N, 2.69.

Complex 2c

Complex **1c** was stirred at room temperature in CH_2Cl_2 (1 mL) for 16 hours at room temperature, during which time a color change from orange to light yellow, reaction mixture was concentrated. Complex **2c** was formed quantitatively as a diastereomeric mixture.

$^{31}P\{^1H\}$ NMR (162 MHz, CD_2Cl_2, offset @ -10 ppm, with ratios): δ = 29.74 (d, *J* = 18.1 Hz, 1.0), 28.35 (d, *J* = 19.3 Hz, 0.5), 19.77 (d, *J* = 18.2 Hz, 1.0), 17.61 (d, *J* = 19.2 Hz, 0.5);

^1H NMR: (400 MHz, CD_2Cl_2, hydride region with ratios): 7.86 – 7.73 (m), 7.64 (d), 7.60 – 7.51 (m), 7.49 (d, *J* = 7.6 Hz), 7.43 (d, *J* = 15.3 Hz), 7.35 (ddd, *J* = 14.3, 7.6, 2.0 Hz), 7.28 – 7.16 (m), 7.14 (s), 7.12 (s), 7.06 – 7.03 (m), 7.03 (s), 6.95 (s), 6.94 (s), 6.91 – 6.81 (m), 6.77 – 6.69 (m), 6.58 (dd, *J* = 15.5, 7.6 Hz), 6.00 (s), 5.55 (s), 4.47 – 4.31 (m), 3.94 – 3.81 (m), 3.25 (m), 2.87 (m), 2.03 (s), 2.01 (s), 1.90 (s), 1.57 (s), 1.30 – 1.18 (m), 1.19 – 1.12 (m), 1.06 (d, *J* = 6.6 Hz), 1.00 (dd, *J* = 6.7, 4.8 Hz), 0.94 (d, *J* = 6.6 Hz), -21.87 (t, *J* = 21.5 Hz, 1.0), -22.93 (t, *J* = 21.1 Hz, 0.5); **HR MS** (CSI^+): *m/z* calcd. for $C_{57}H_{69}IrN_2O_5P_2S_2$ $[M+H]^+$: 1181.3830, observed: 1181.3726; **Anal. Calcd.** for $C_{57}H_{69}IrN_2O_5P_2S_2$: C, 58.00; H, 5.89; N, 2.37, found: C, 57.82; H, 5.93, N, 2.31.

VT-NMR of diastereo-pure complex 2a

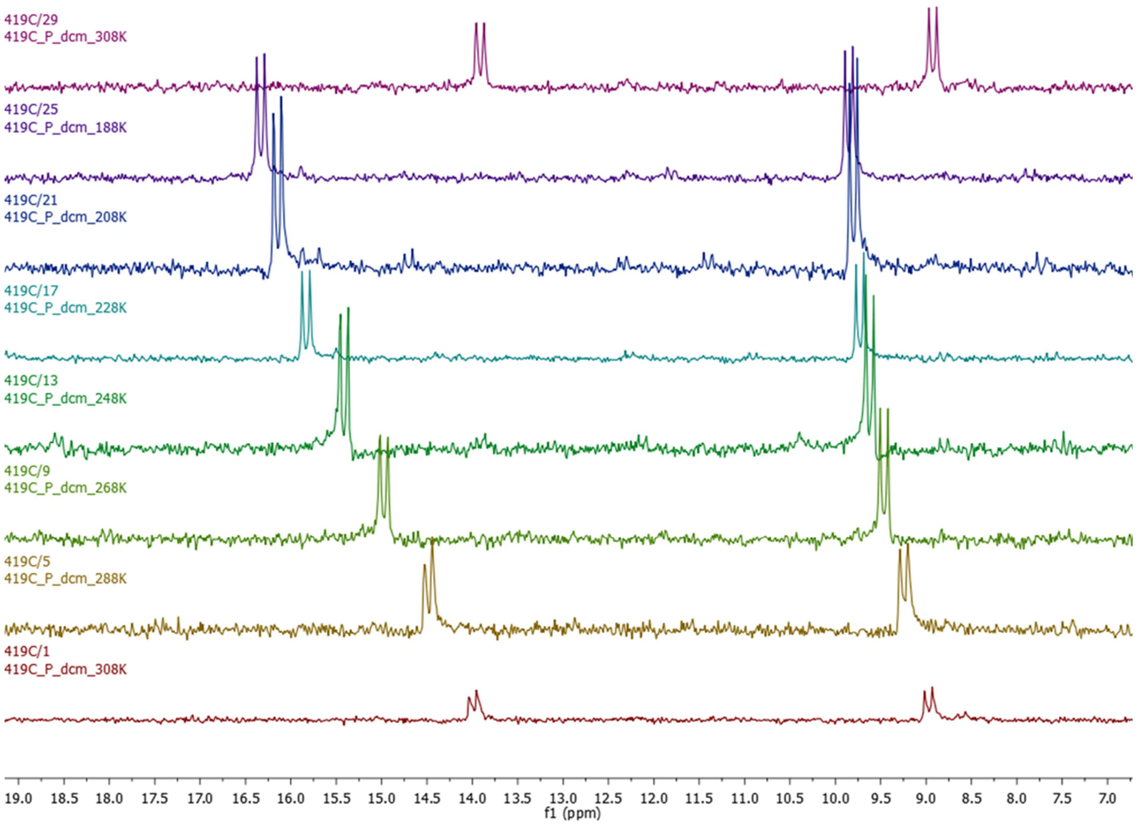

S1. Temperature dependence of ^{31}P NMR of complex **2a** (diastereo-pure) in CD_2Cl_2.

VT-NMR of diastereo-pure complex 2a with 1eq. HCOOH

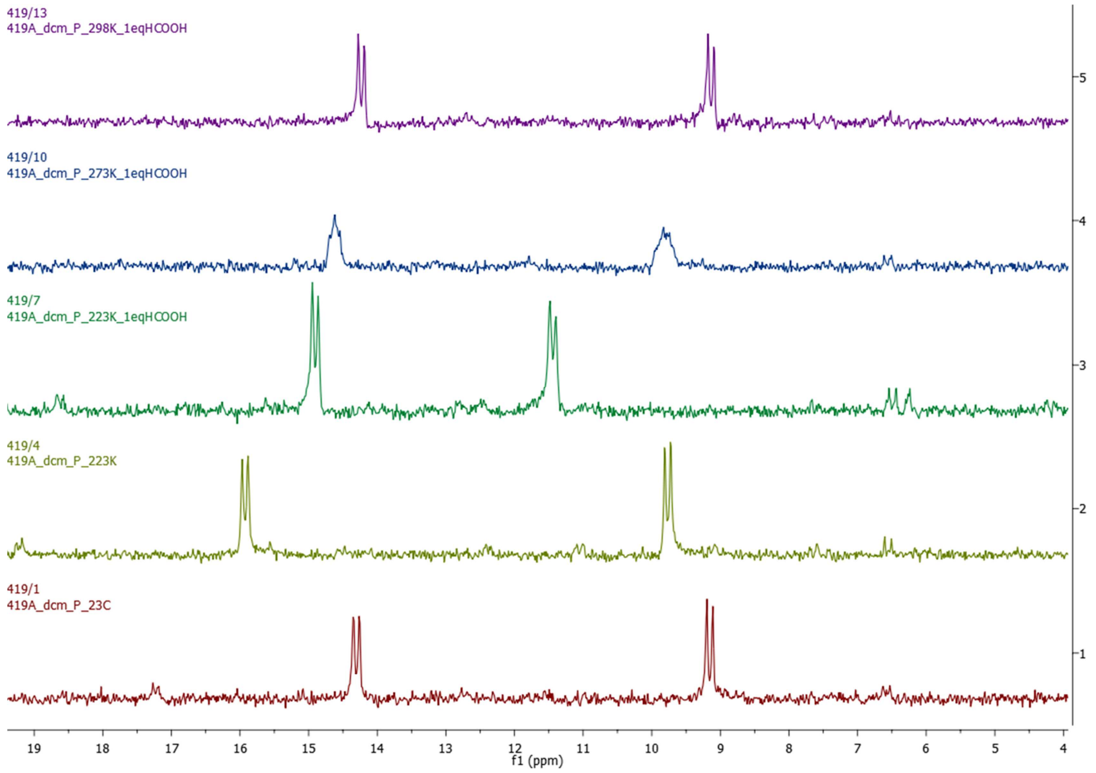

S2. VT ^{31}P NMR of complex **2a** (diastereo-pure) with 1eq. of HCOOH in CD_2Cl_2.

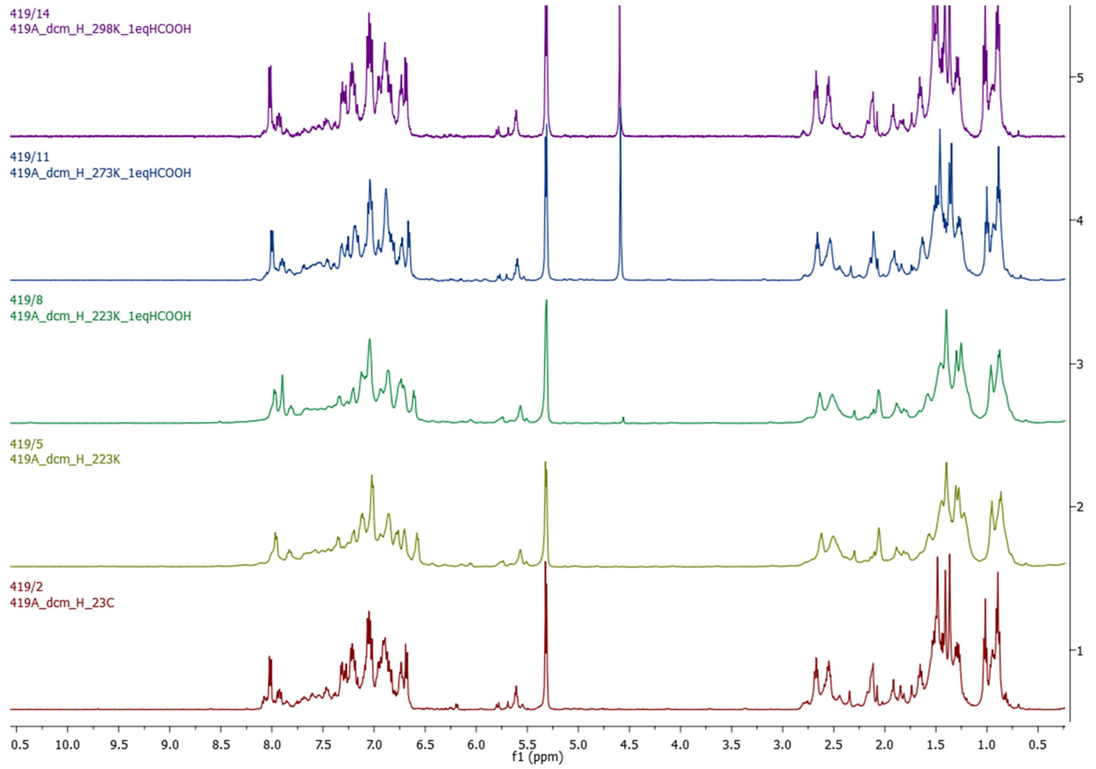

S3. VT ^{1}H NMR of complex **2a** (diastereo-pure) with 1eq. of HCOOH in CD_2Cl_2.

Crystal structure of 2c

$C_{57}H_{71}IrN_2O_6P_2S_2 \cdot CH_2Cl_2 \cdot 0.5(C_4H_{10}O)$, Fw = 1320.40, colourless needle, 0.52 x 0.12 x 0.11 mm^3, triclinic, $P\bar{1}$ (no. 2), a = 11.6106(3), b = 17.2668(6), c = 18.6161(4) Å, α = 113.198(2), β = 95.475(2), γ = 105.211(1)°, V = 3226.02(16) $Å^3$, Z = 2, D_x = 1.359 g/cm^3, μ = 2.31 mm^{-1}. The crystal appeared to cracked into two fragments and was consequently integrated with two orientation matrices using the Eval15 software[S8]. 50934 Reflections were measured on a Bruker Kappa ApexII diffractometer with sealed tube and Triumph monochromator (λ = 0.71073 Å) up to a resolution of $(\sin \theta/\lambda)_{max}$ = 0.65 $Å^{-1}$ at a temperature of 150(2) K. Absorption correction and scaling based on multiple measured reflections was performed with TWINABS[S9] (0.59-0.75 correction range). 14853 Reflections were unique (R_{int} = 0.018), of which 14073 were observed [$I>2\sigma(I)$]. The structure was solved with the program SHELXT[S10] and refined with SHELXL-2013[S11] against F^2 of all reflections. Non-hydrogen atoms were refined freely with anisotropic displacement parameters. Hydrogen atoms of the metal complex were located in difference-Fourier maps, and in the solvent molecules included in calculated positions. All hydrogen atoms were refined with a riding model. The diethyl ether molecule was refined with partial occupancy. 718 Parameters were refined with 30 restraints (for displacement parameters in the partially occupied diethyl ether). R1/wR2 [$I > 2\sigma(I)$]: 0.0273 / 0.0777. R1/wR2 [all refl.]: 0.0293 / 0.0788. S = 1.073. Residual electron density between -1.14 and 3.46 e/$Å^3$. Geometry calculations and checking for higher symmetry was performed with the PLATON program[S12].

CCDC 1020151 contains the supplementary crystallographic data for this paper. These data can be obtained free of charge from The Cambridge Crystallographic Data Centre via www.ccdc.cam.ac.uk/data_request/cif.

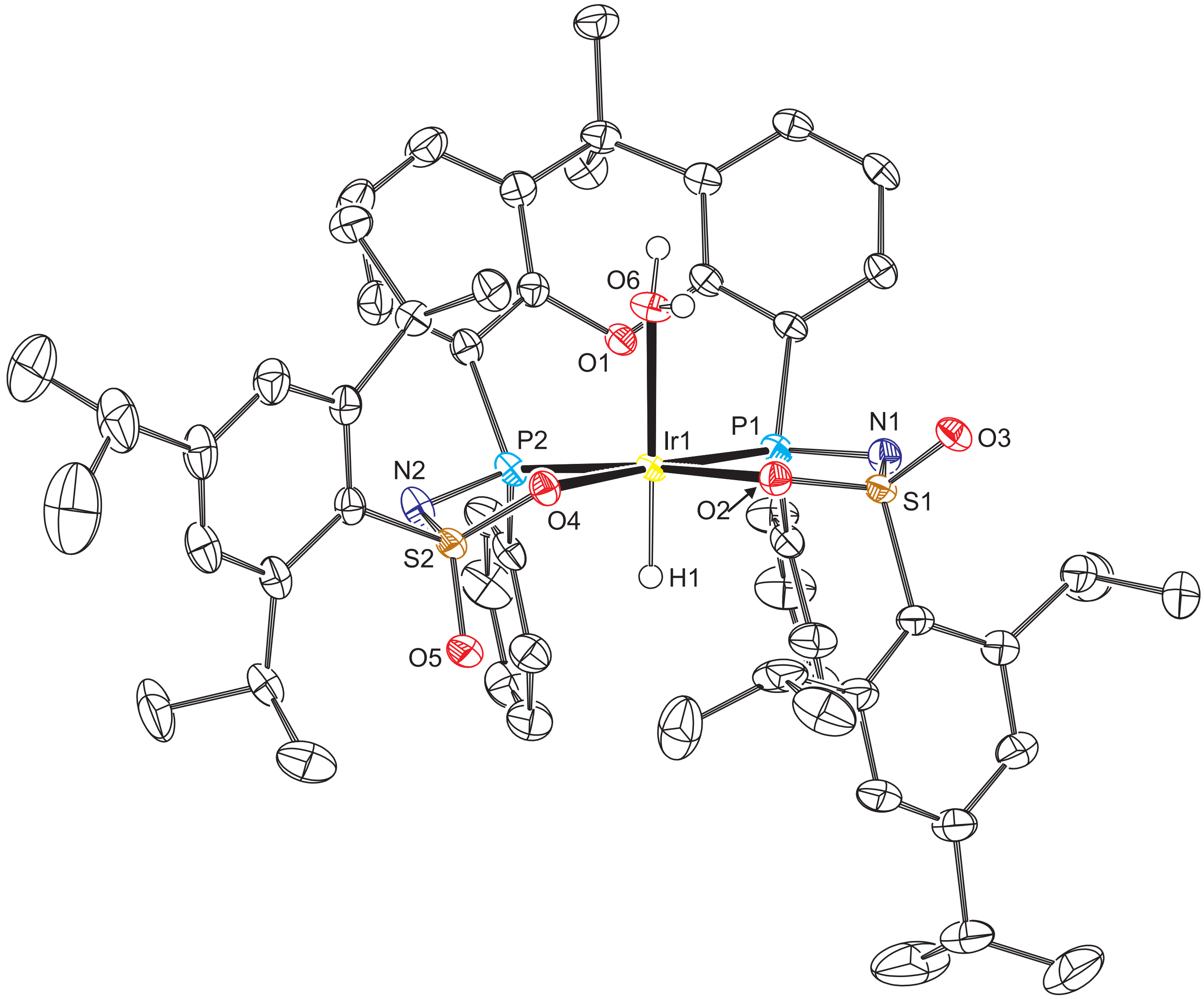

Figure S4: Displecement ellipsoid plot of **2c** in the crystal (50% probability level). Dichloromethane and diethyl ether solvent molecules and C-H hydrogen atoms in the metal complex are omitted for clarity.

Diastereomeric structure used in calculations

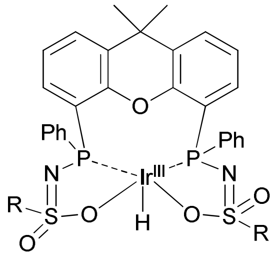

R = phenyl

Energies and imaginairy frequencies of calculated structures

Structure	SCF	imaginairy frequency
1	-3497,75372	
2	-3497,78130	
3I	-3687,64937	
3II	-3687,61999	-81,820000
3III	-3498,96500	
3IV	-3498,92829	-1143,810059
3IV'	-3498,93657	-454,299988
4I	-3687,66088	
4II	-3687,62186	-336,519989
4III	-3498,95775	
4IV	-3498,91508	-1369,430054
4IV'	-3688,81986	-919,200012
5I	-3687,67416	
5II	-3687,65150	
5III	-3687,61982	-230,509995
5IV	-3498,93008	
5V	-3498,92683	-78,129997
6I	-3687,66344	
6II	-3687,64222	
6III	-3687,63084	-163,179993
7I	-3687,65060	
7II	-3687,62691	
7III	-3687,61419	-237,690020
7IV	-3498,94369	
8I	-3687,64763	
2-CF_3	-4172,20640	
5I-CF_3	-4361,59084	
5II-CF_3	-4362,07590	
5III-CF_3	-4362,04346	-239,220001
6I-CF_3	-4362,08918	
6II-CF_3	-4362,06680	

6III-CF_3	-4362,05498	-138,169998
2-CH_3	-3576,44453	
5I-CH_3	-3766,33745	
5II-CH_3	-3766,31484	
5III-CH_3	-3766,28365	-229,729996
6I-CH_3	-3766,32655	
6II-CH_3	-3766,30561	
6III-CH_3	-3766,29462	-166,929996

Energy profiles of structures 3I and 4I

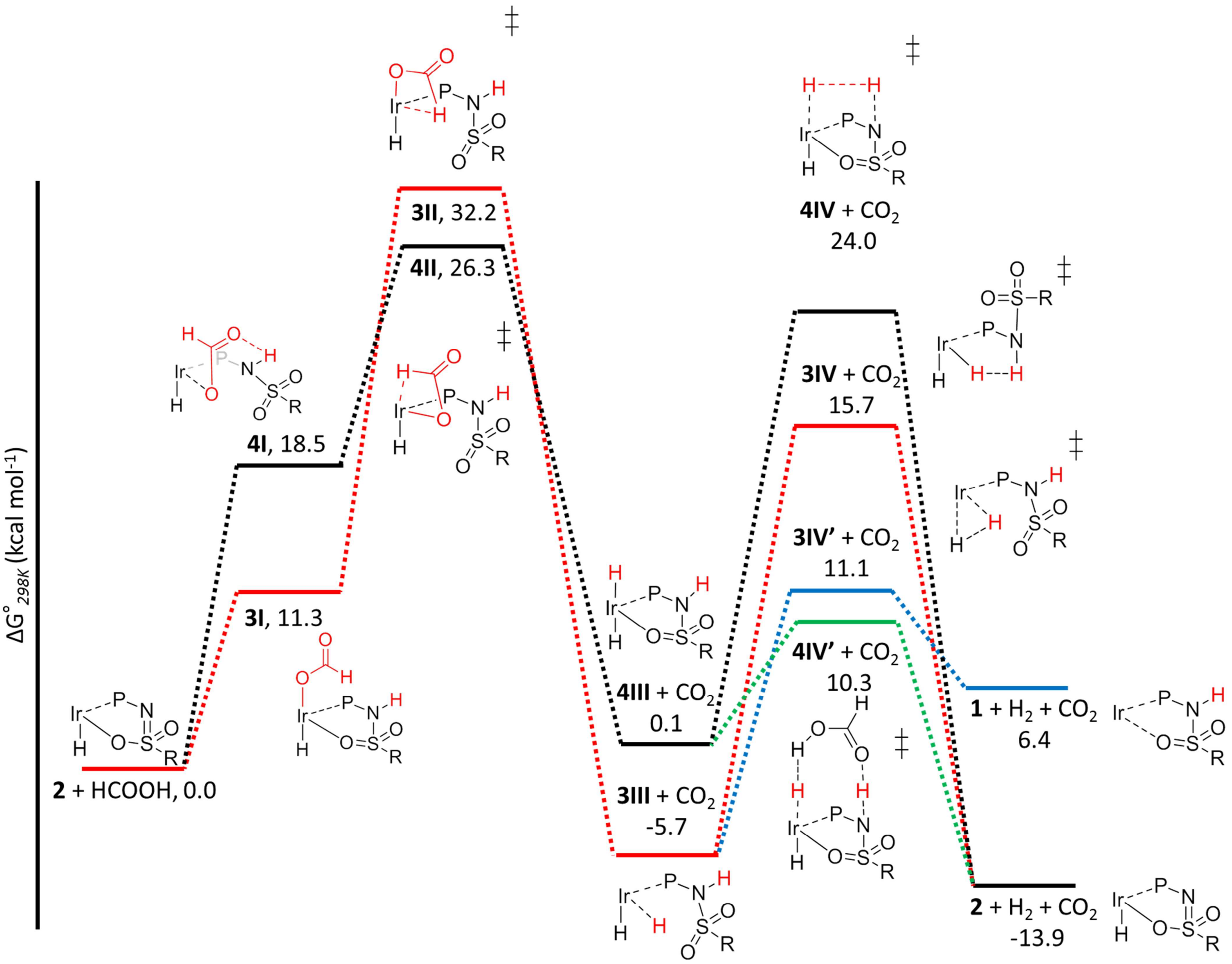

Figure S5. Potential energy surfaces (DFT, BP86, def2-TZVP) for dehydrogenation of formic acid by **3I and 4I** (ΔG^{o}_{298K} in kcal mol^{-1}).

Energy profiles of structures 5I and 6I

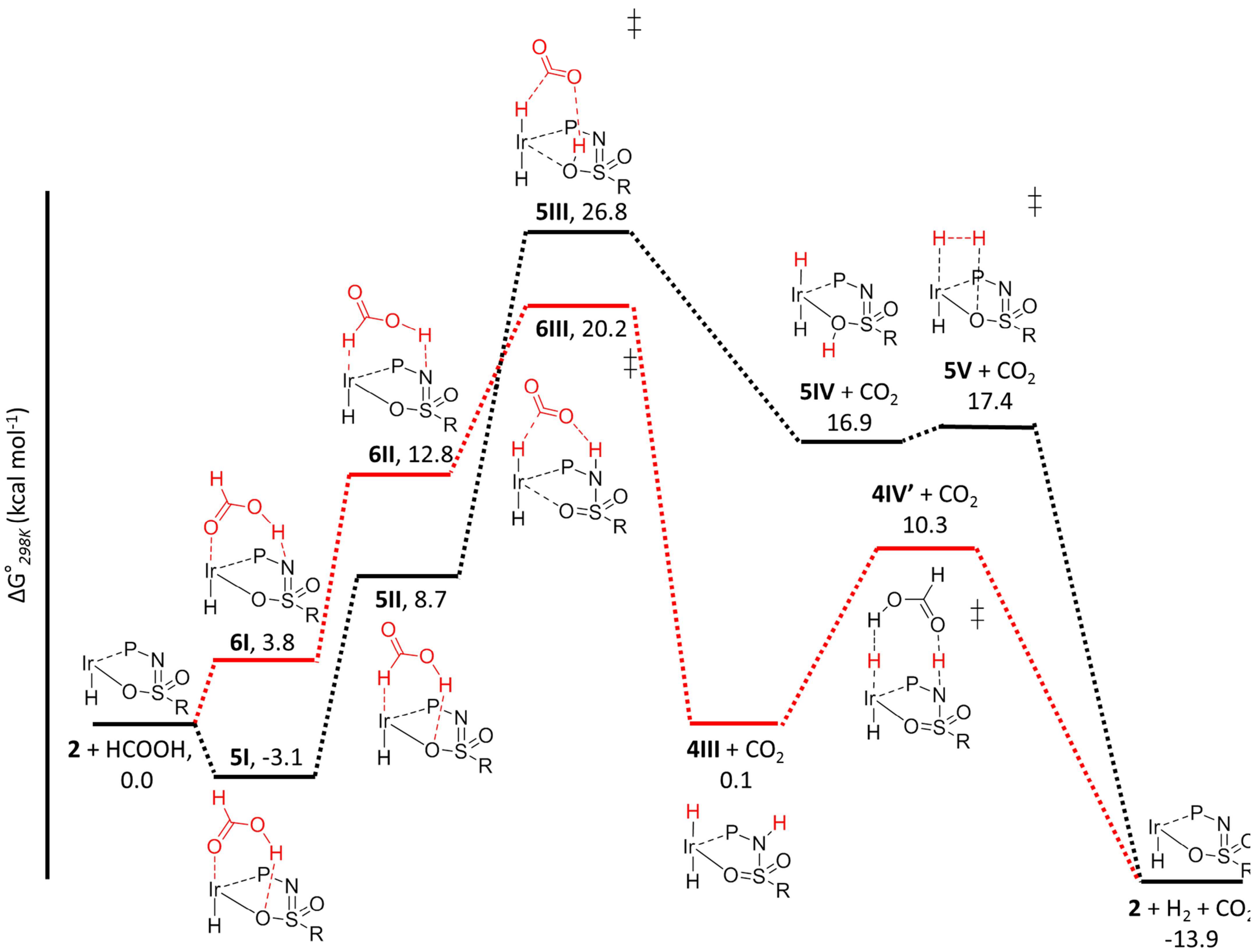

Figure S6. Potential energy surfaces (DFT, BP86, def2-TZVP) for dehydrogenation of formic acid by **5I and 6I** (ΔG°_{298K} in kcal mol^{-1}).

Energy profile of structure 7I

Rearrangement of HCOOH in **7I** to orient the substrate in the right position for direct hydride-transfer to yield structure **7II** was found to be endergonic by 23.5 kcal mol^{-1}. The transition state (**7III**) of the direct hydride-transfer toward the dihydride structure **7IV** was found to be significantly higher (29.9 kcal mol^{-1}) than for the axial structures **5III** and **6III**. Similar to the transition states previously found (**5III** and **6III**) hydrogen-bonding interactions were also observed in **7III**. The release of H_2 has been described above, for complete energy profile of **7I** see supporting information. Starting from complex **8I**, rearrangement of HCOOH to enable direct hydride-transfer led to an unstable species and no transition state could be identified.

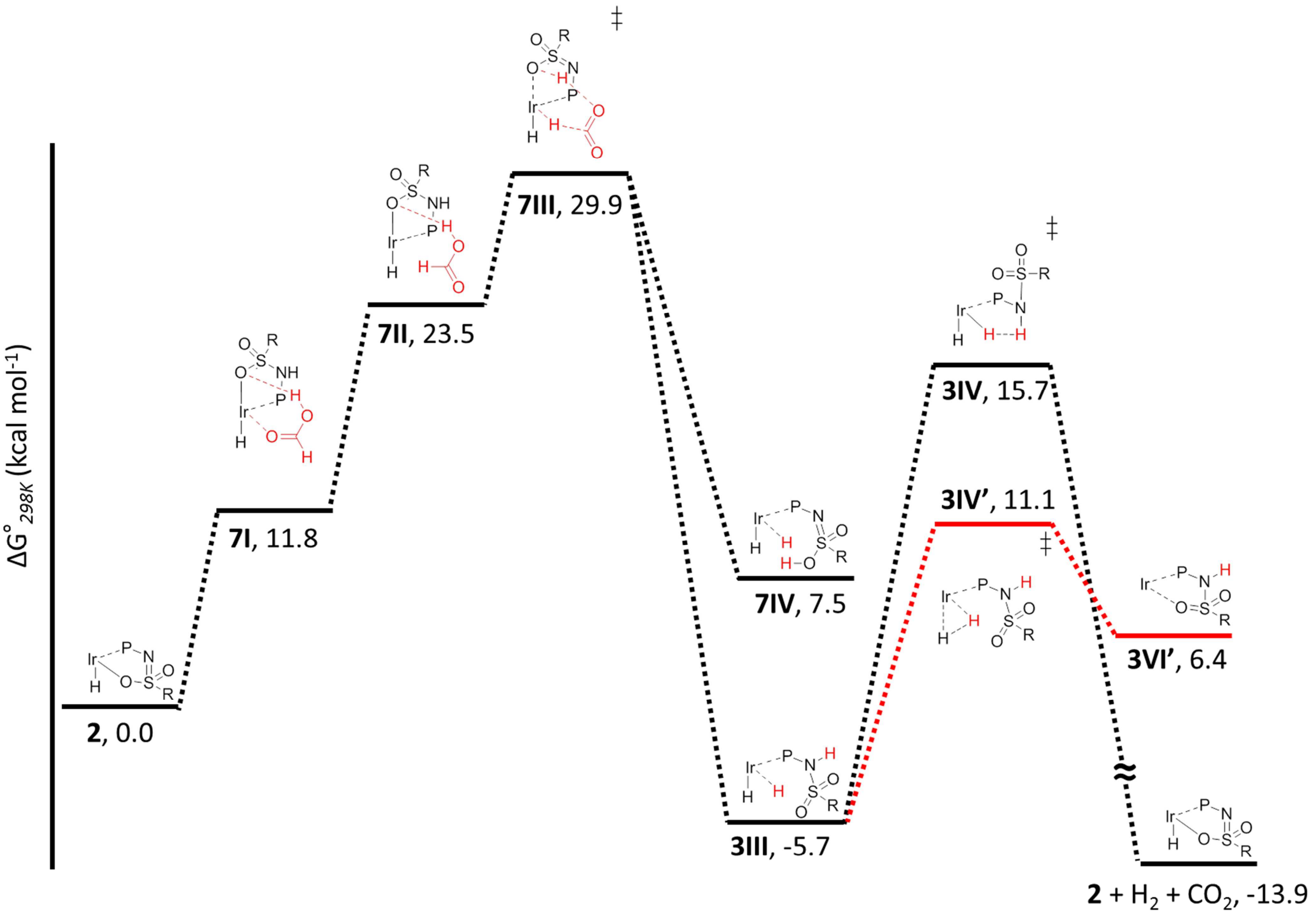

Figure S7. Potential energy surfaces (DFT, BP86, def2-TZVP) for dehydrogenation of formic acid by **7I** (ΔG^{o}_{298K} in kcal mol^{-1}).

Energy profile with CF_3 and CH_3 sustituents

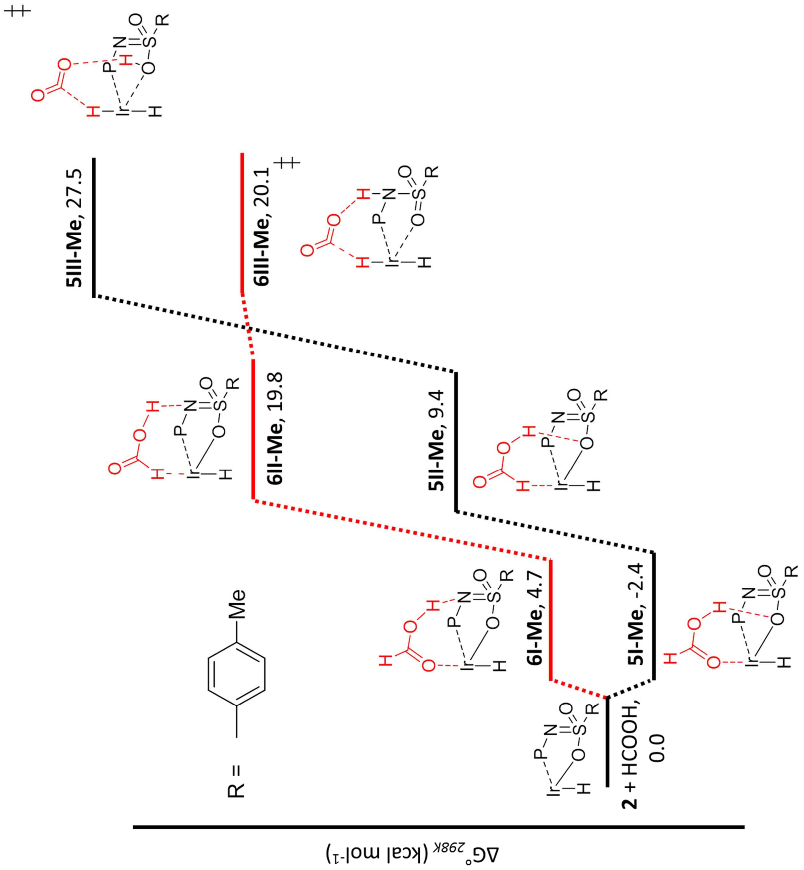

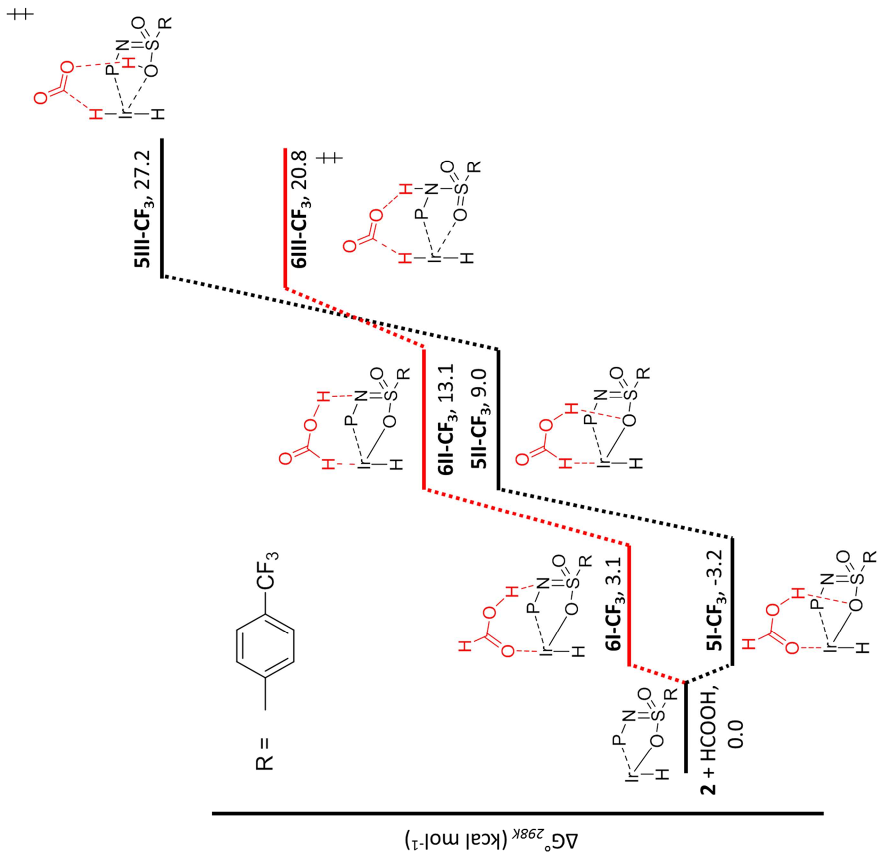

Figure S8. Potential energy surfaces (DFT, BP86, def2-TZVP) for dehydrogenation of formic acid by with CF_3 and CH_3 substituents (ΔG^{o}_{298K} in kcal mol^{-1}).

^{31}P NMR ligand La

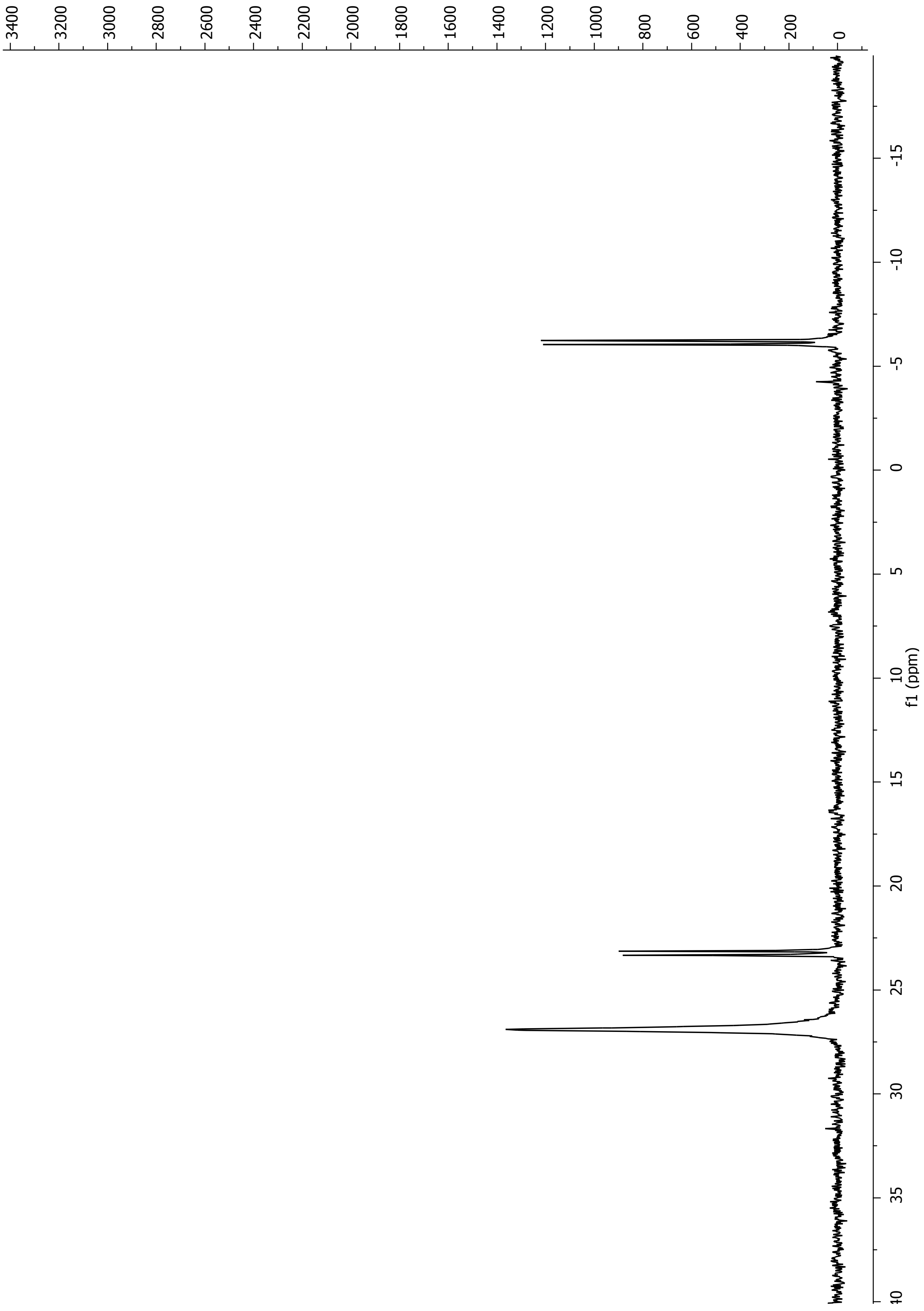

^{1}H NMR ligand La

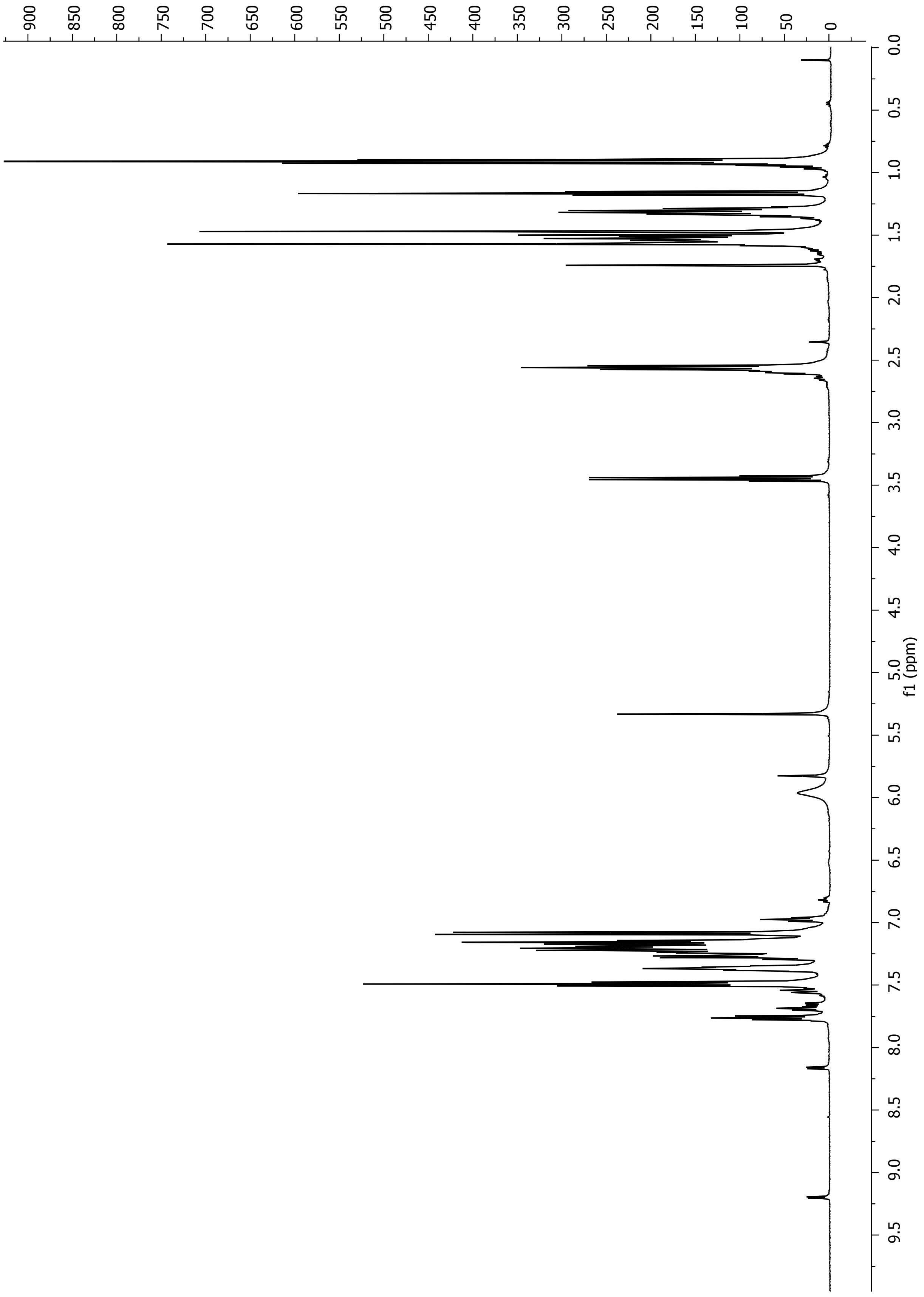

^{13}C NMR ligand La

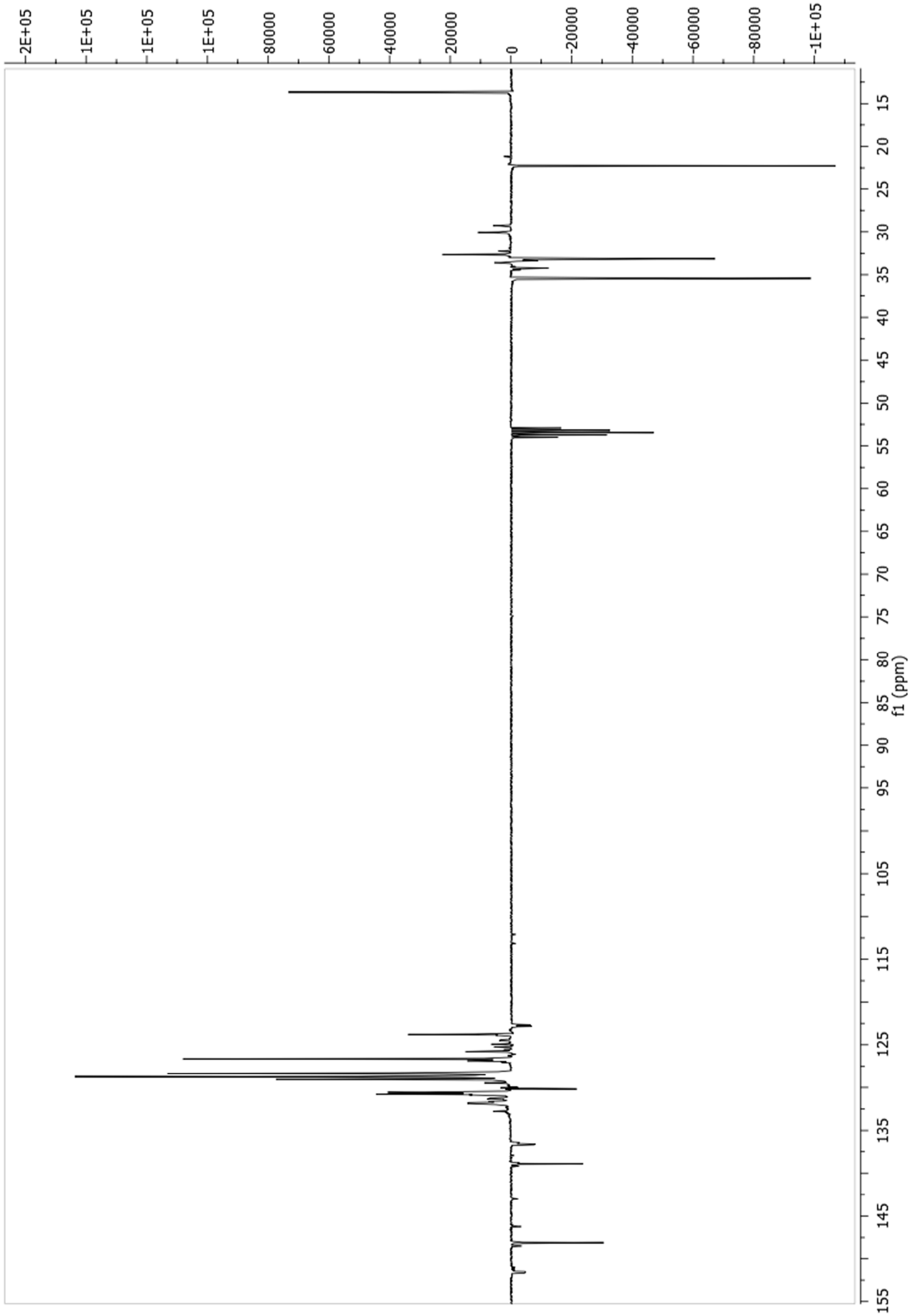

^{31}P NMR ligand Lb

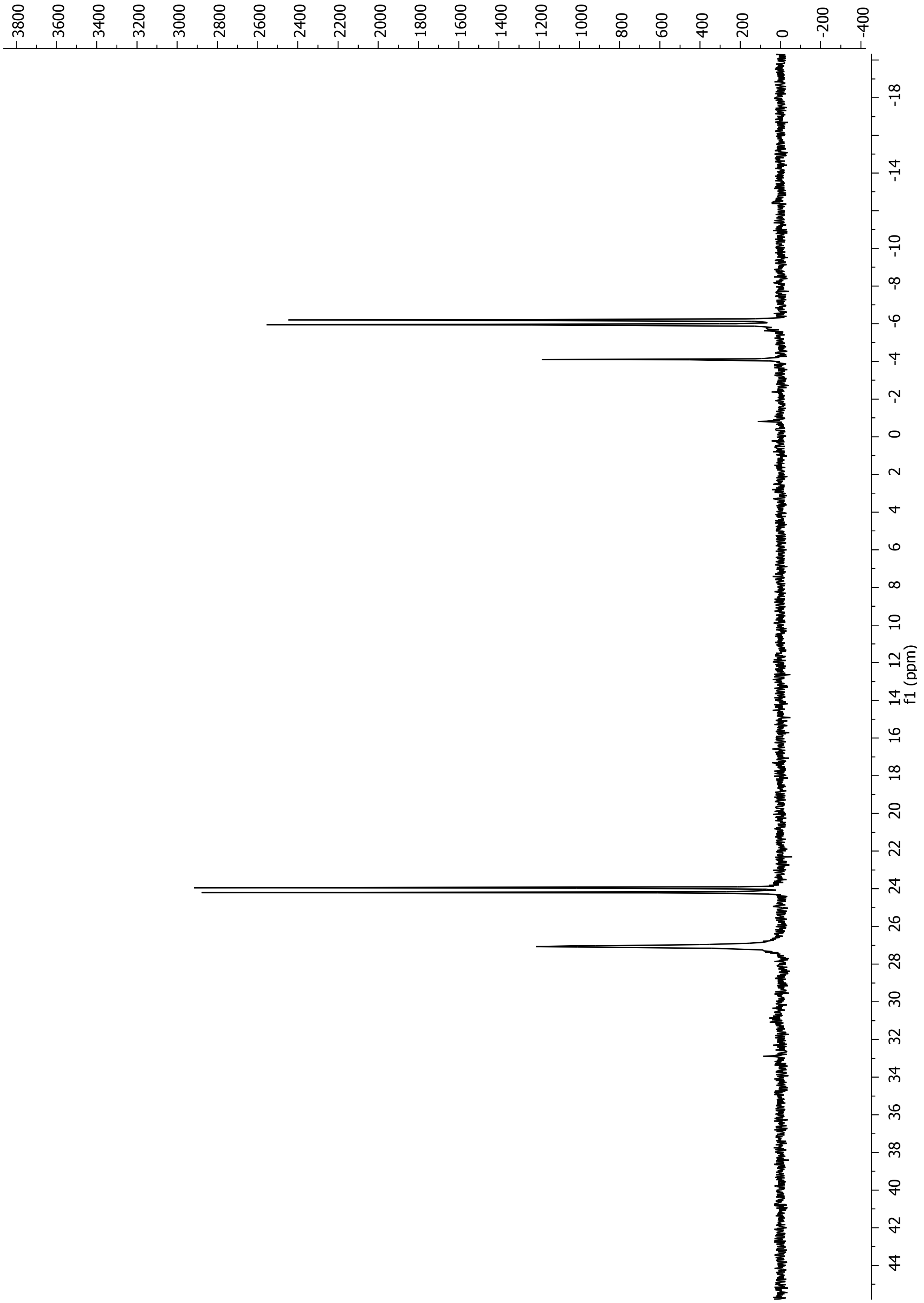

^{1}H NMR ligand Lb

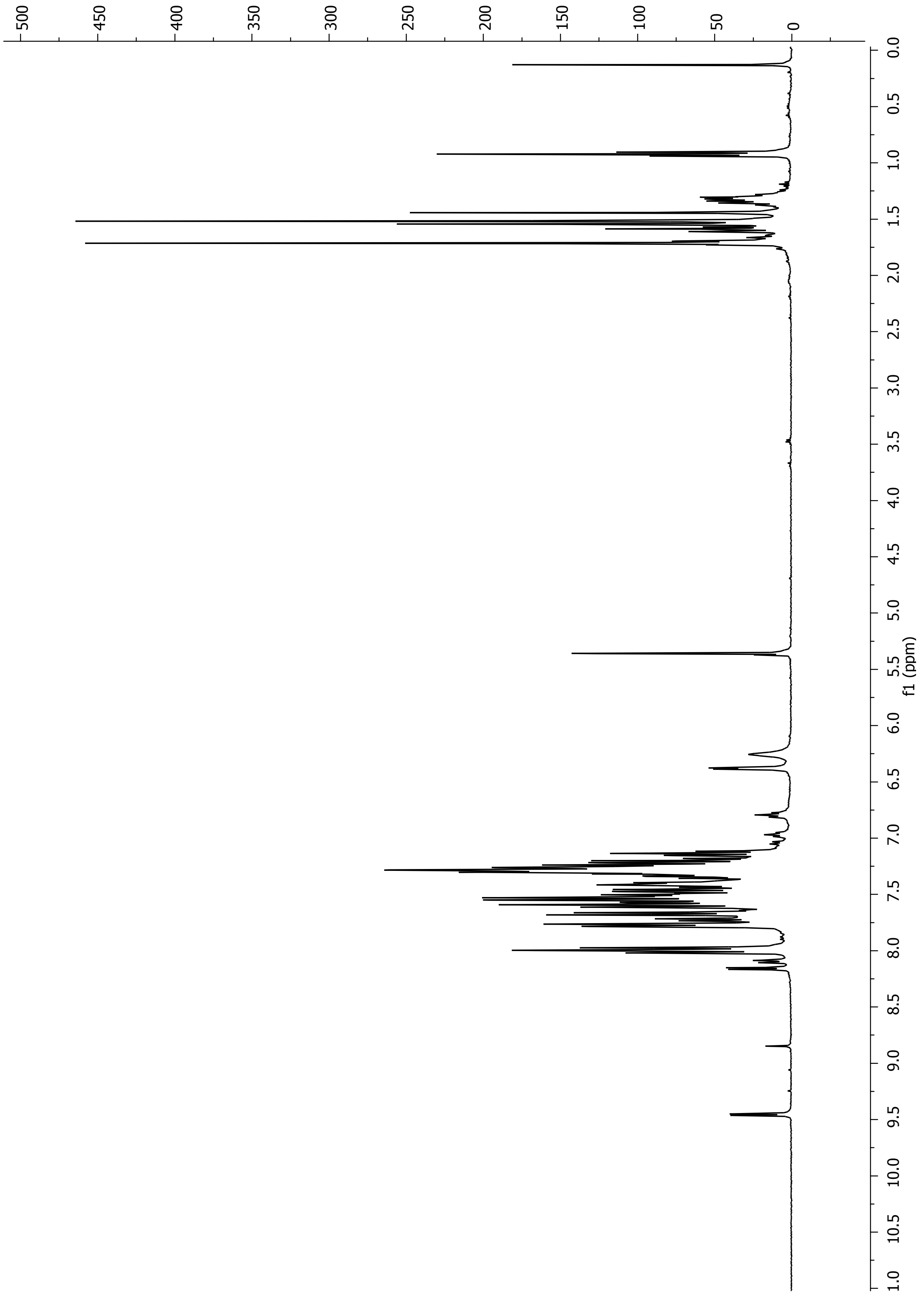

^{13}C NMR ligand Lb

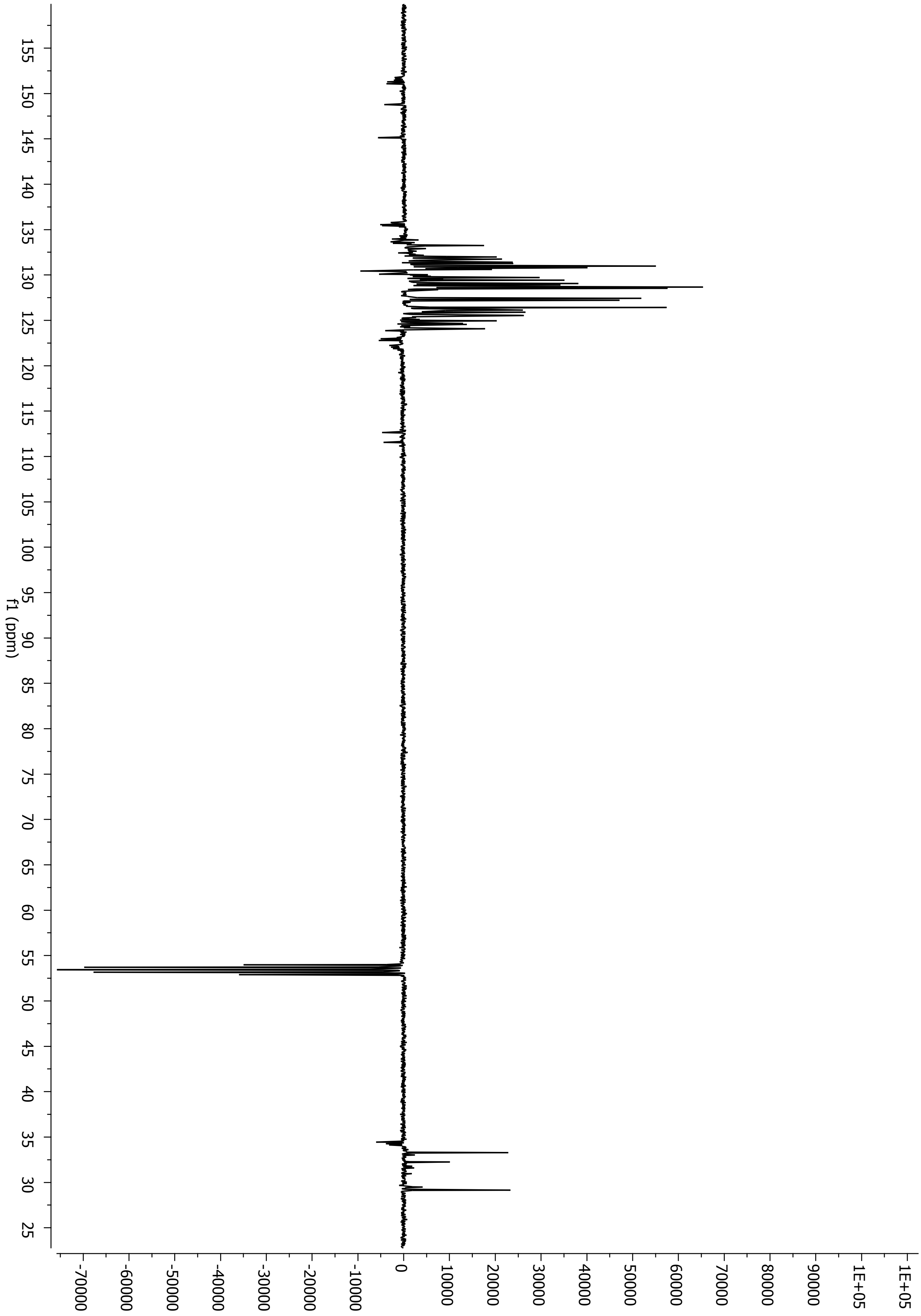

^{19}F NMR ligand Lb

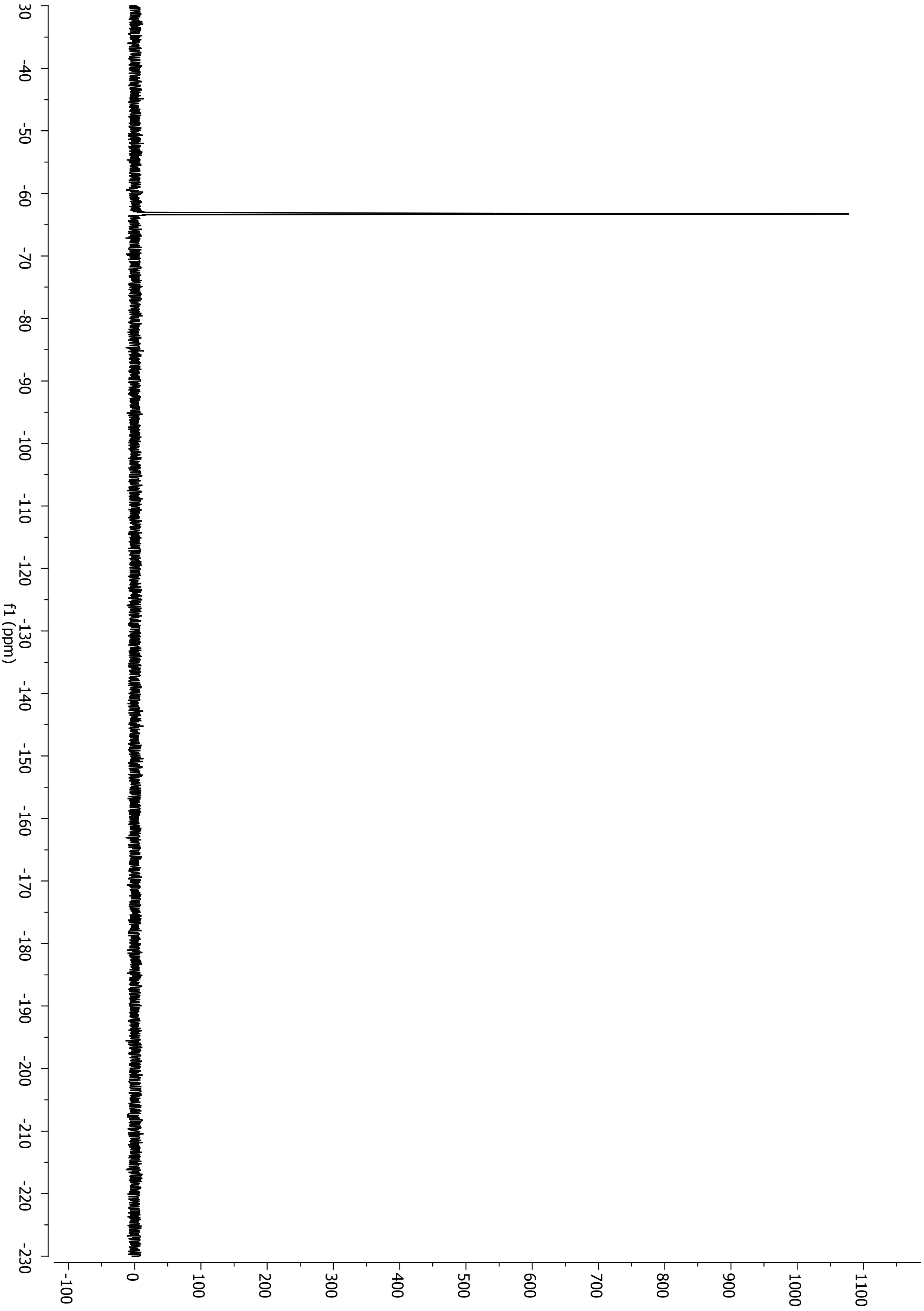

^{31}P NMR ligand Lc

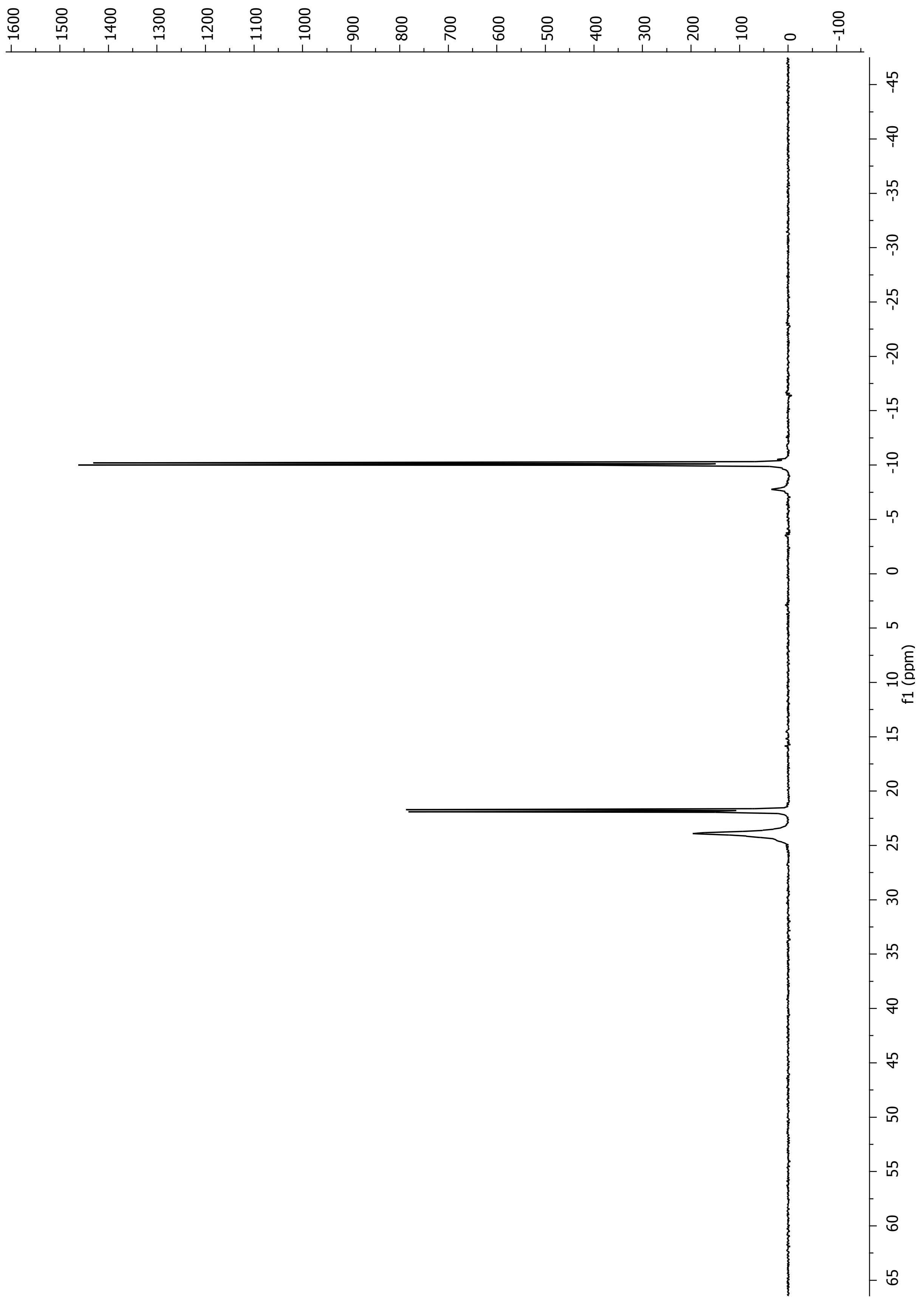

^{1}H NMR ligand Lc

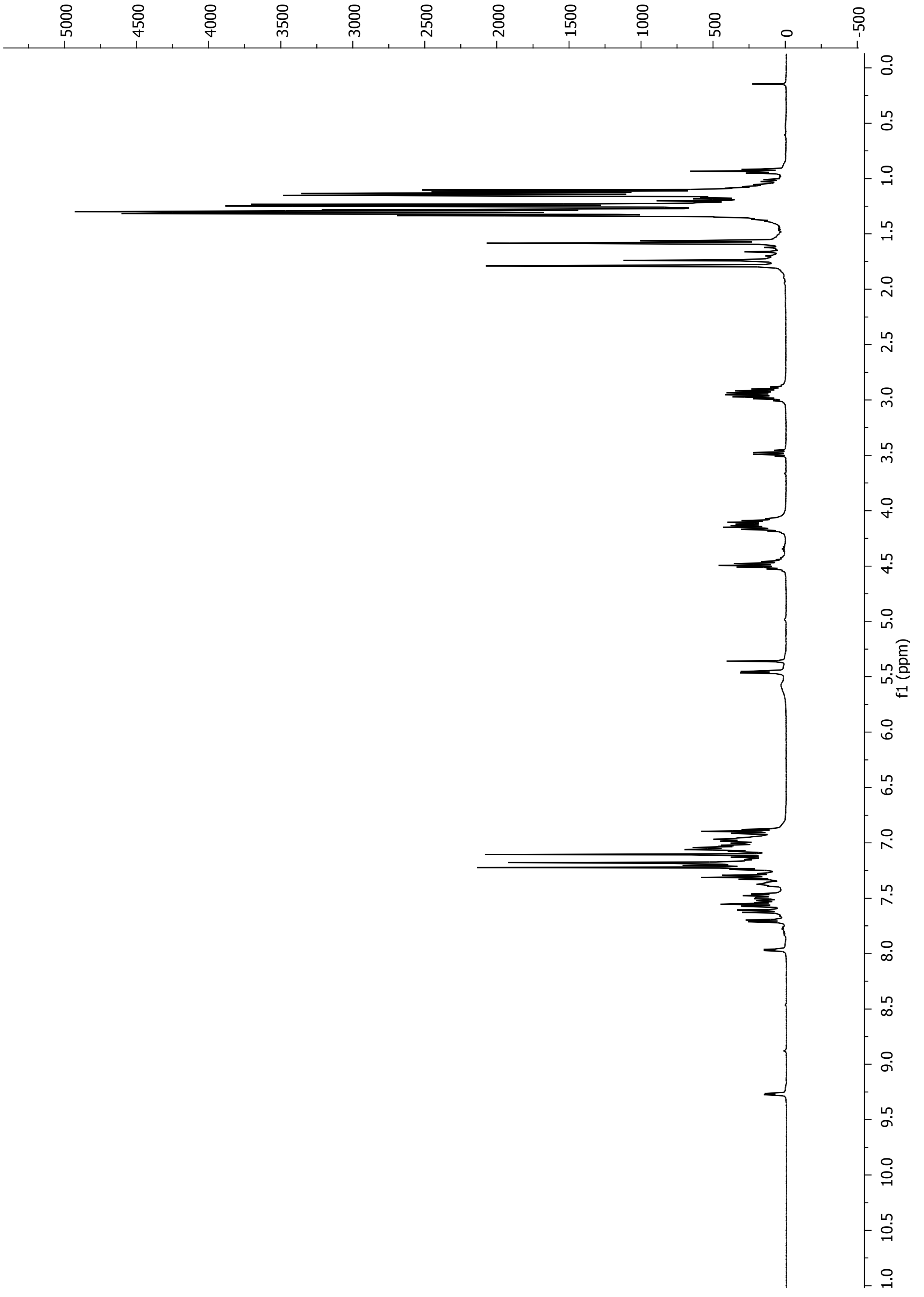

^{13}C NMR ligand Lc

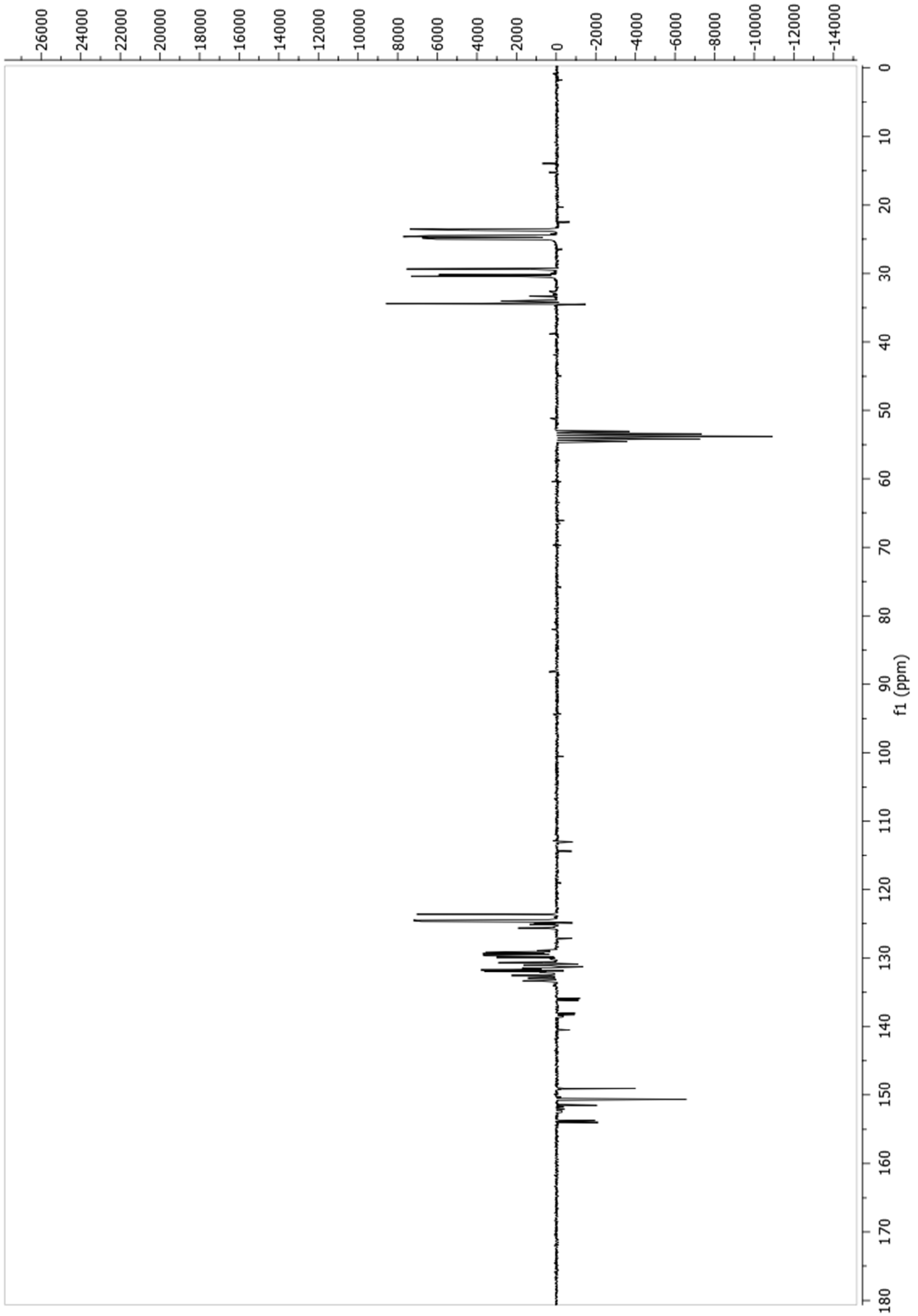

^{31}P NMR 2a

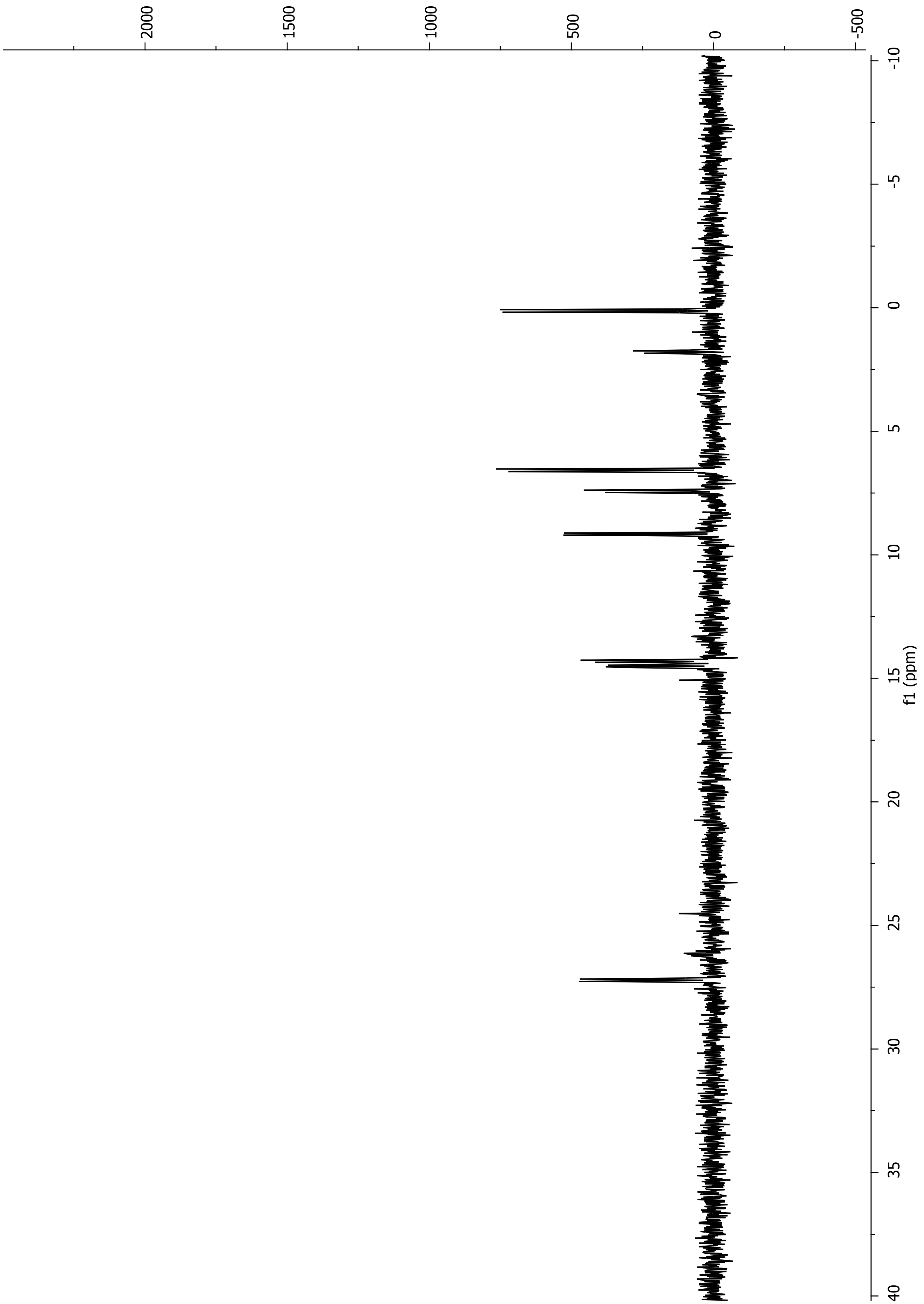

^{1}H NMR 2a

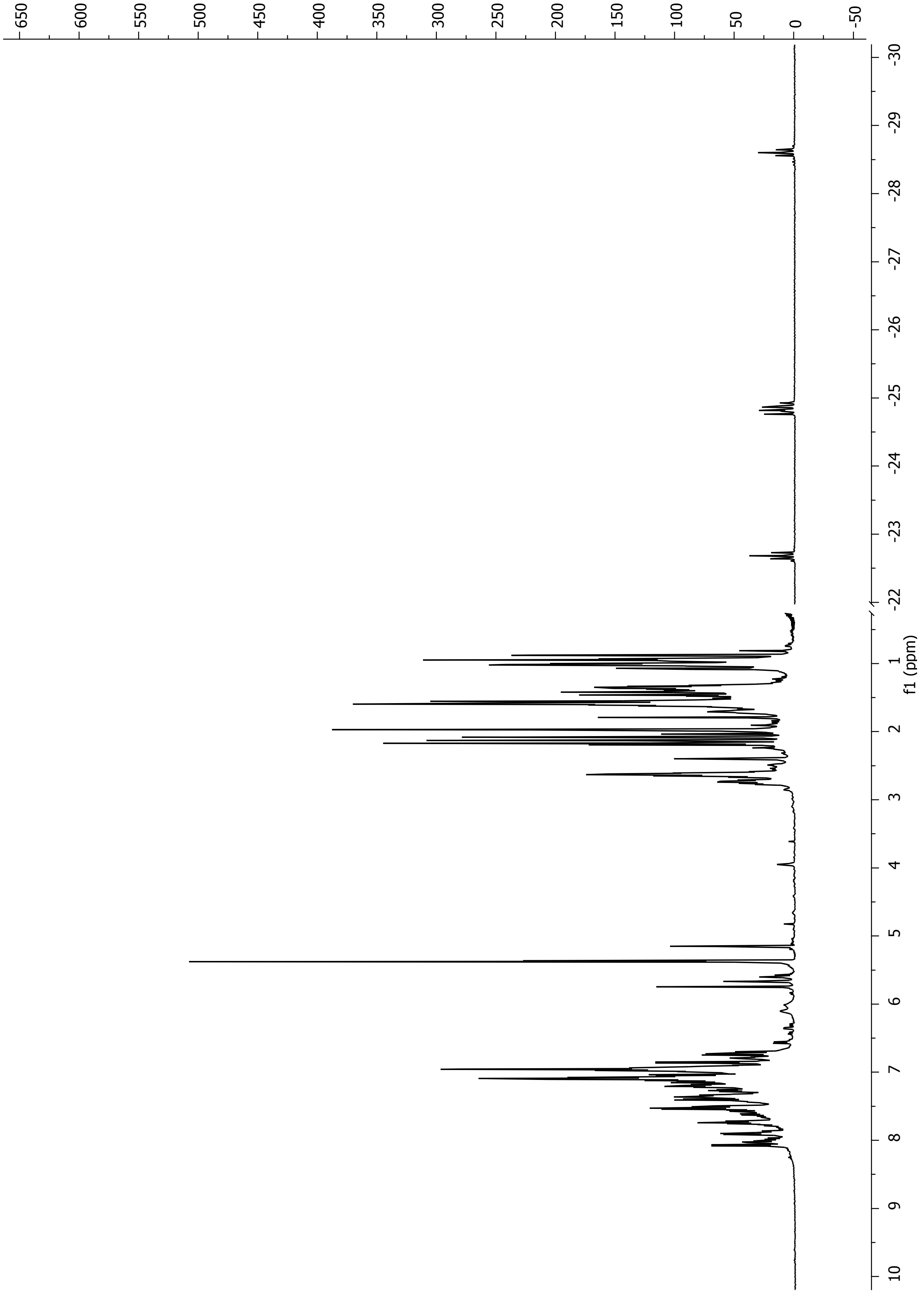
650 600 550 500 450 400 350 300 250 200 150 100 50 0 -50
-30 -29 -28 -27 -26 -25 -24 -23 -22
1 2 3 4 5 6 7 8 9 10
f1 (ppm)

^{31}P NMR 2b

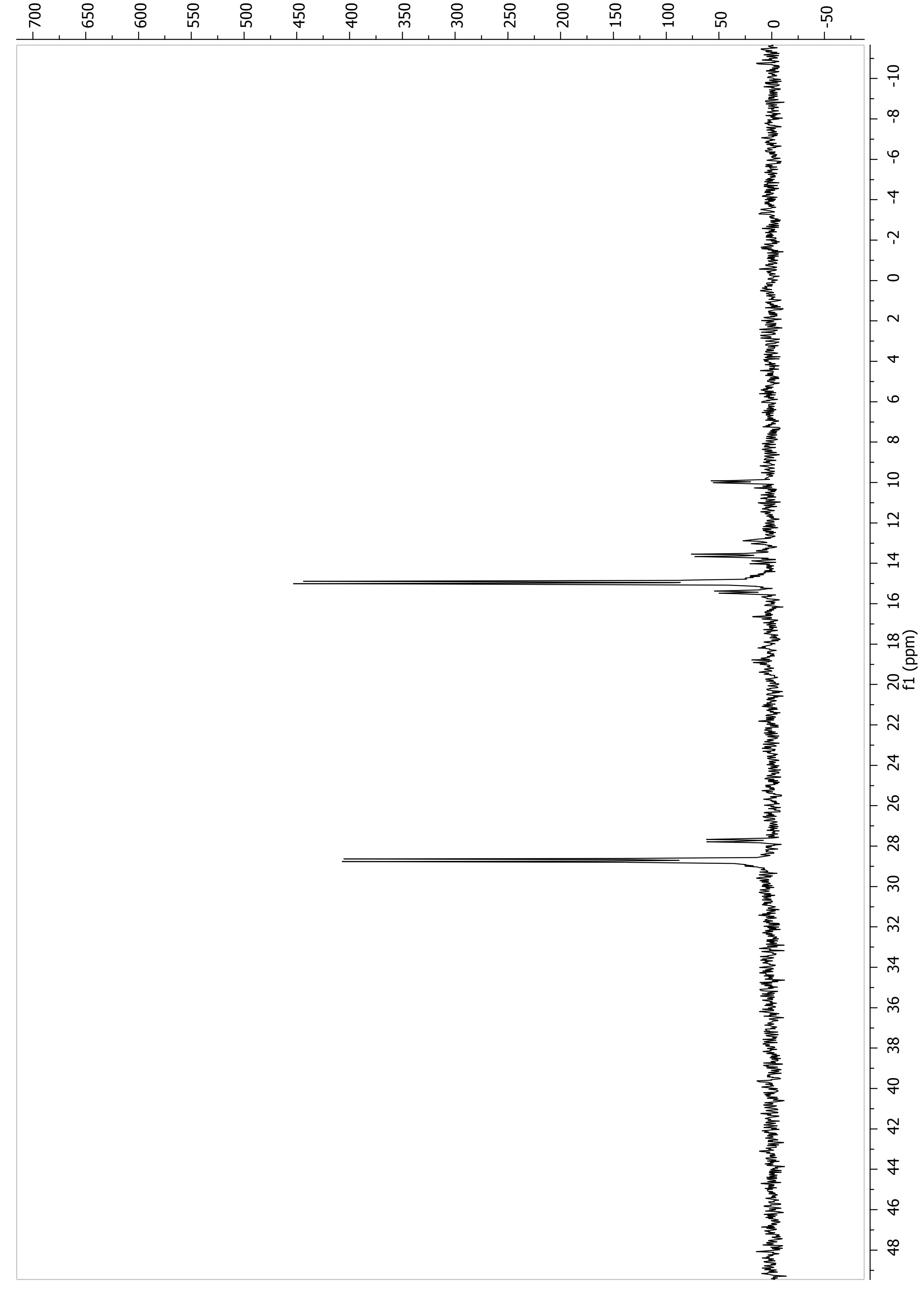

^{1}H NMR 2b

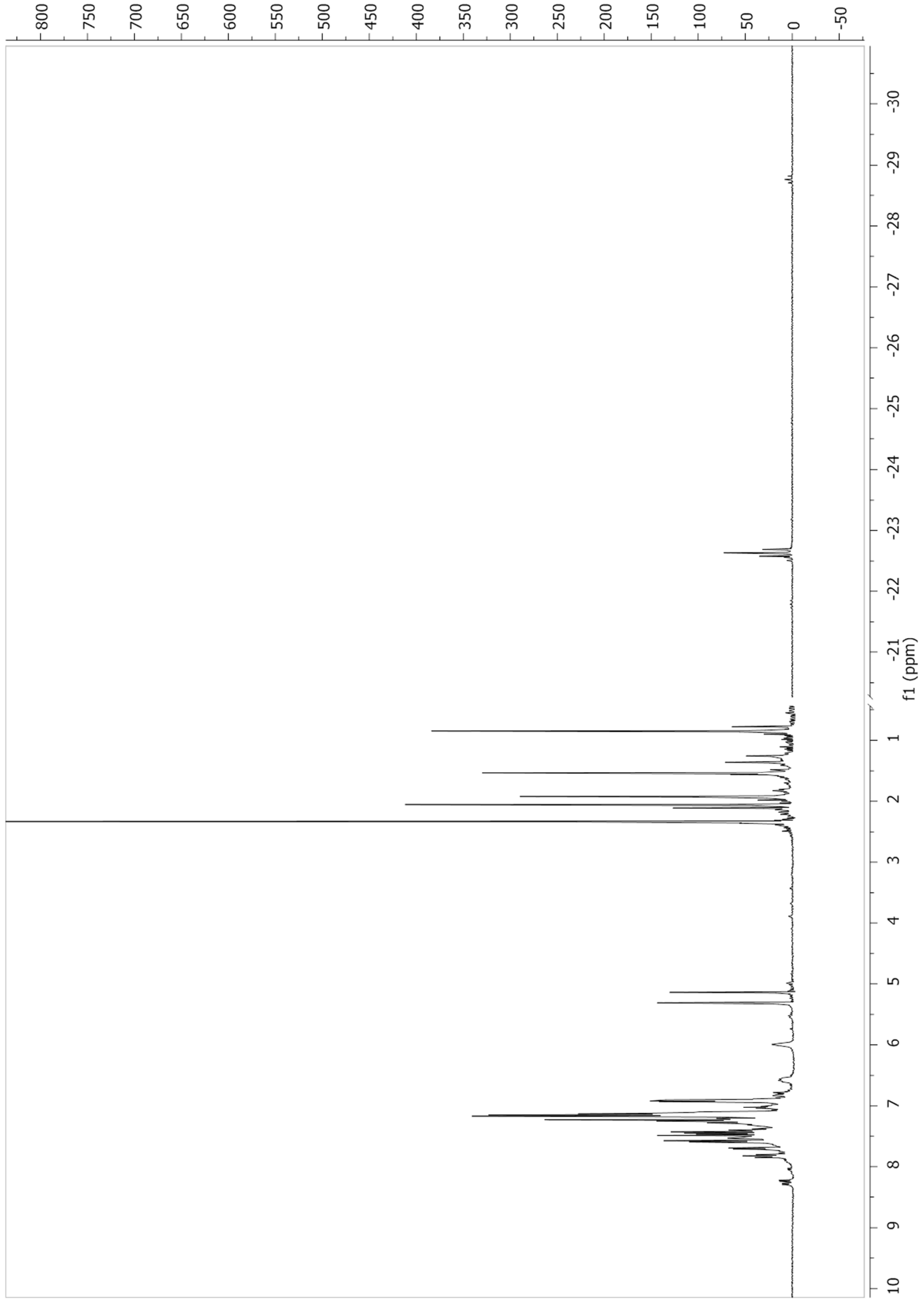

^{19}F NMR 2b

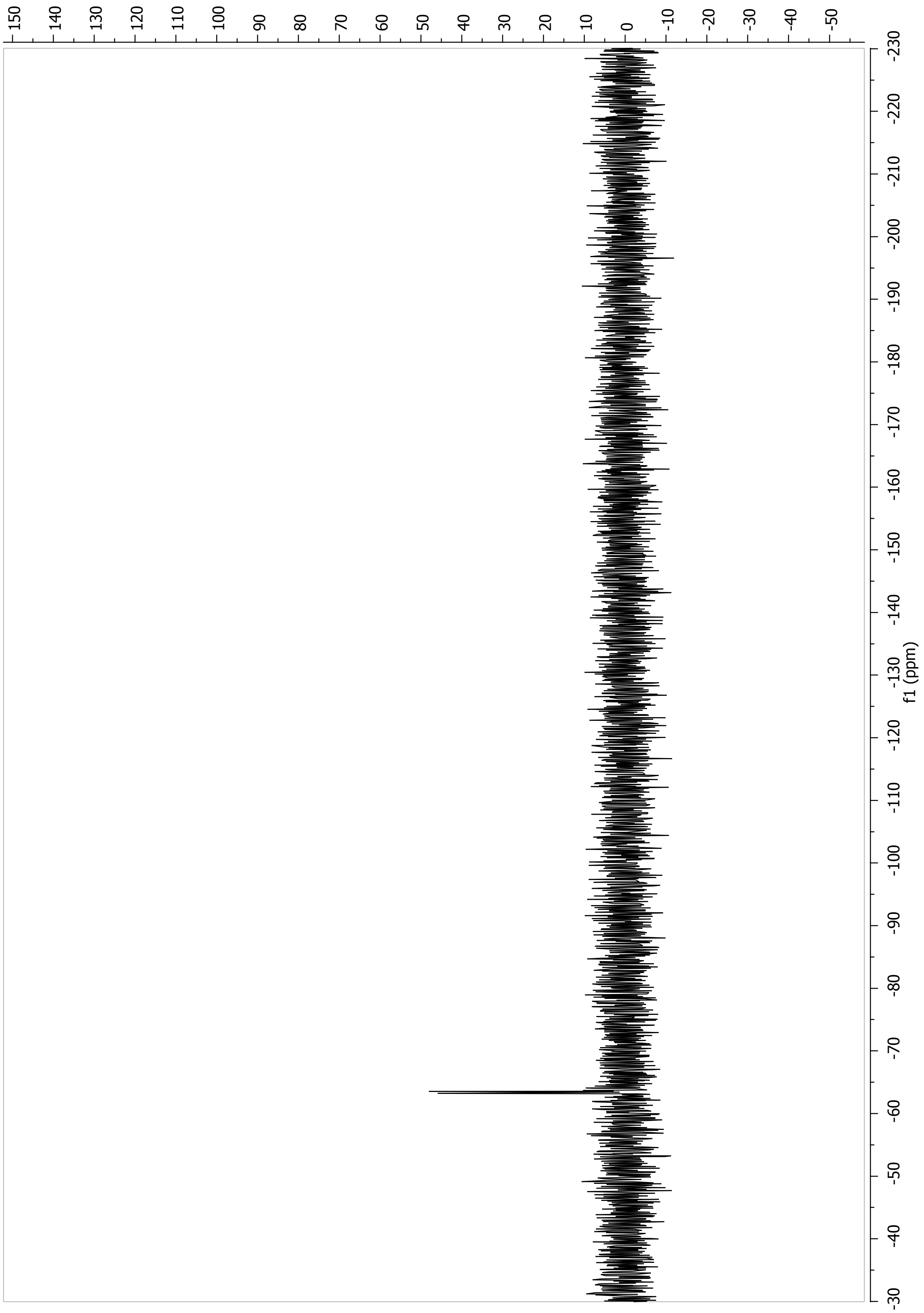

^{31}P NMR 2c

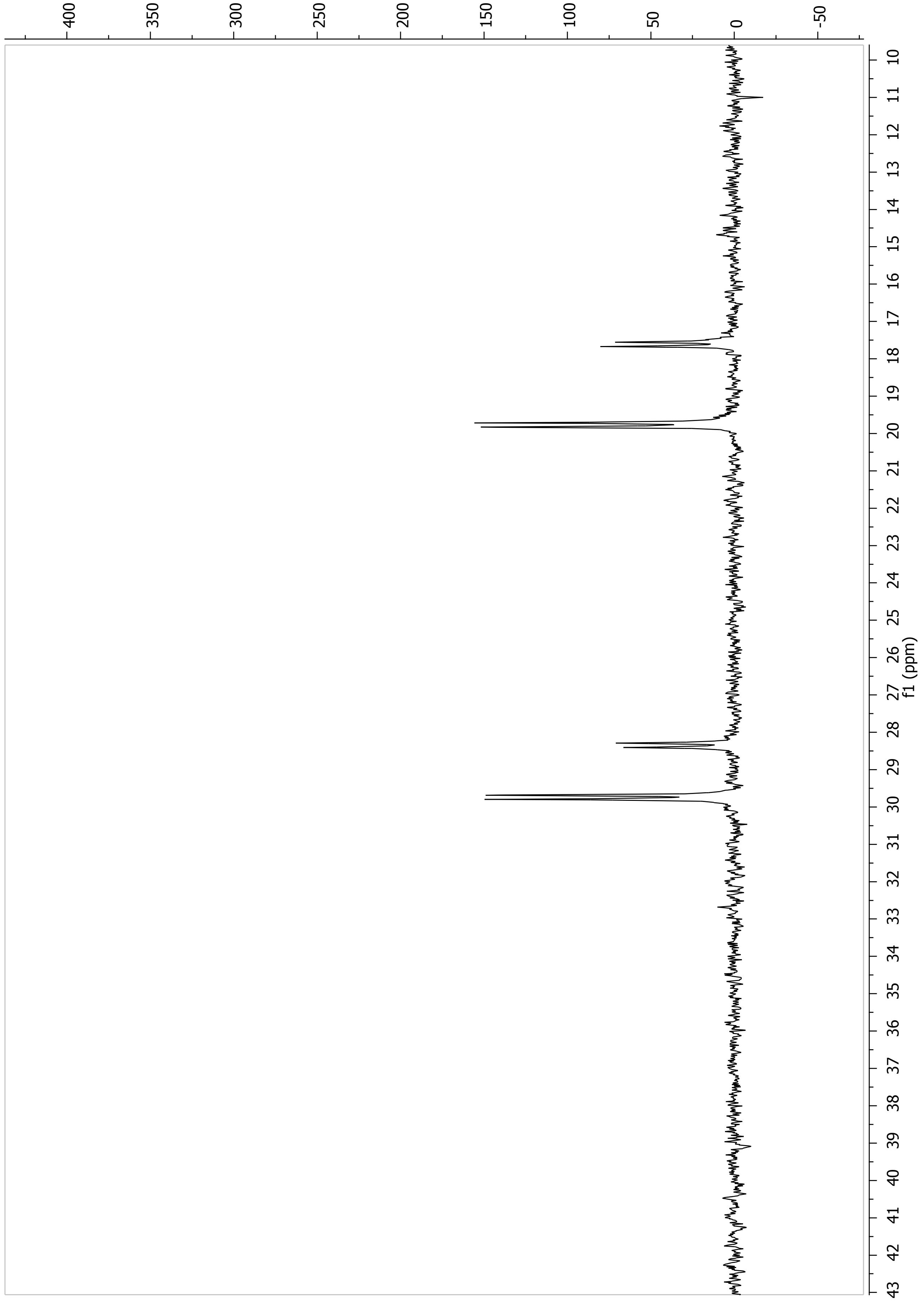

^{1}H NMR 2c

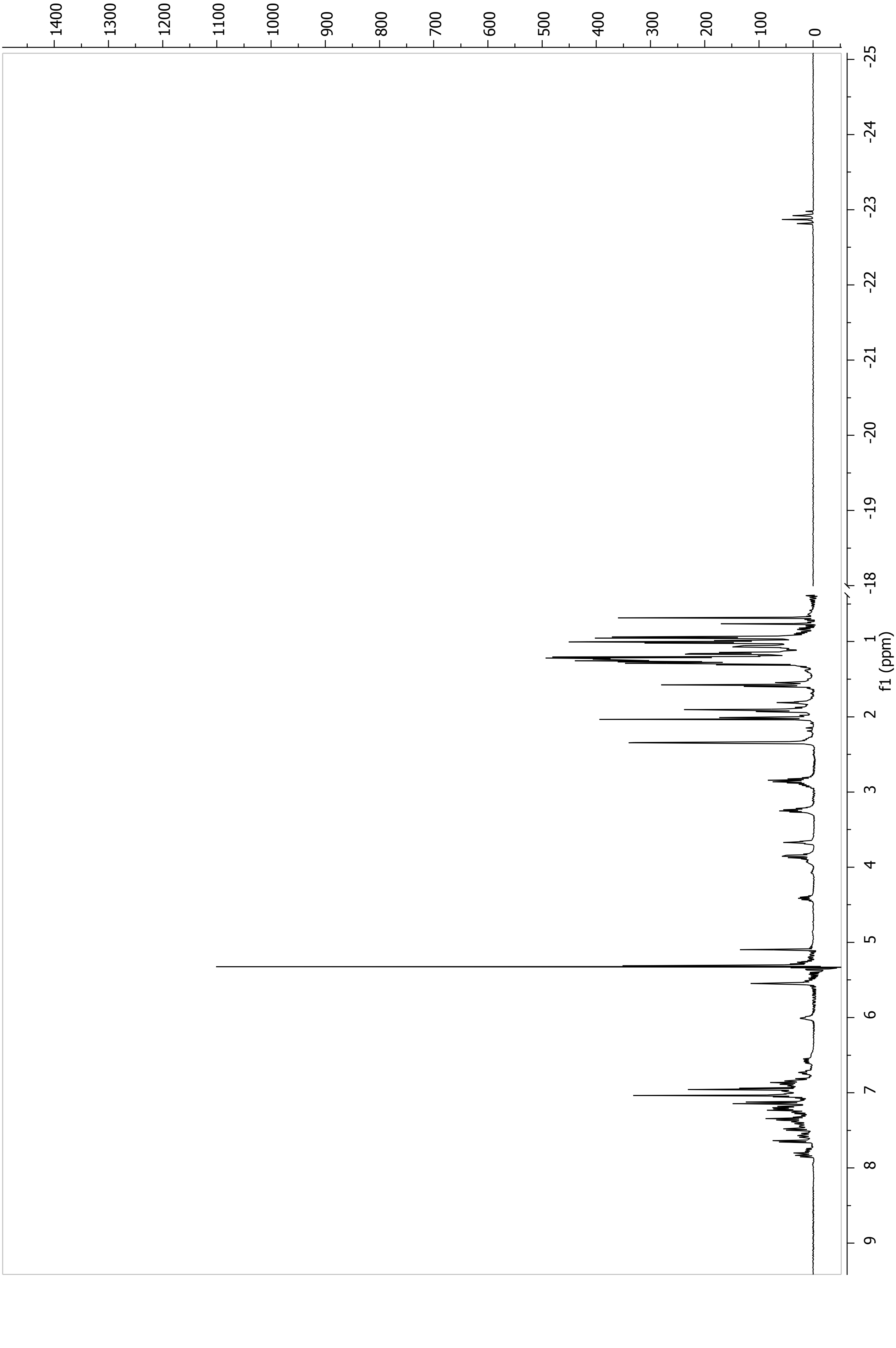

References

[S1] S. Fukuzumi, T. Kobayashi, T. Suenobu, *J. Am. Chem. Soc.* 2010, **132**, 1496-1497.

[S2] R. Ahlrichs, Turbomole Version 5, 2002, Theoretical Chemistry Group, University of Karlsruhe.

[S3] PQS version 2.4, 2001, Parallel Quantum Solutions, Fayettevile, Arkansas (USA); the Baker optimizer is available separately from PQS upon request: I. Baker, *J. Comput. Chem*. 1986, **7**, 385-395.

[S4] P. H. M. Budzelaar, *J. Comput. Chem*. 2007, **28**, 2226-2236.

[S5] (a) A. D. Becke, *Phys. Rev. A*, 1988, **38**, 3098–3100. (b) J. P. Perdew, *Phys. Rev. B*, 1986, **33**, 8822- 8824.

[S6] M. Sierka, A. Hogekamp, R. Ahlrichs, *J. Chem. Phys.* 2003, **118**, 9136-9148.

[S7] A. Schaefer, H. Horn, R. Ahlrichs, *J. Chem. Phys*. 1992, **97**, 2571–2577.

[S8] A. M. M. Schreurs, X. Xian, L. M. J. Kroon-Batenburg, *J. Appl. Cryst.* 2010, **43**, 70-82.

[S9] G. M. Sheldrick (1999). TWINABS, Universität Göttingen, Germany.

[S10] G. M. Sheldrick (2013). SHELXT, Universität Göttingen, Germany.

[S11] G. M. Sheldrick, *Acta Cryst.* 2008, **A64**, 112-122.

[S12] A.L. Spek, *Acta Cryst.* 2009, D**65**, 148-155.
